# Functionalization of Carbon Nanotubes and Graphene Derivatives with Conducting Polymers and Their Applications in Dye-Sensitized Solar Cells and Supercapacitors

**DOI:** 10.3390/polym16010053

**Published:** 2023-12-22

**Authors:** Mirela Văduva, Teodora Burlănescu, Mihaela Baibarac

**Affiliations:** 1 National Institute of Materials Physics, Atomistilor Street, No 405 A, 077125 Magurele, Romania; teodora.burlanescu@infim.ro (T.B.); barac@infim.ro (M.B.); 2 Faculty of Physics, University of Bucharest, Atomistilor Street, No 405, 077125 Magurele, Romania

**Keywords:** solar cell, functionalization, graphene, carbon nanotube, conducting polymer, composites, power conversion efficiency, supercapacitors, capacitance

## Abstract

Recent progress concerning the development of counter electrode material (CE) from the dye-sensitized solar cells (DSSCs) and the electrode material (EM) within supercapacitors is reviewed. From composites based on carbon nanotubes (CNTs) and conducting polymers (CPs) to their biggest competitor, namely composites based on graphene or graphene derivate (GD) and CPs, there are many methods of synthesis that influence the morphology and the functionalization inside the composite, making them valuable candidates for EM both inside DSSCs and in supercapacitors devices. From the combination of CPs with carbon-based materials, such as CNT and graphene or GD, the perfect network is created, and so the charge transfer takes place faster and more easily. Inside composites, between the functional groups of the components, different functionalizations are formed, namely covalent or non-covalent, which further provide the so-called synergic effect. Inside CPs/CNTs, CNTs could play the role of template but could also be wrapped in a CP film due to π–π coupling enhancing the composite conductivity. Active in regenerating the redox couple I^−^/I_3_^−^, the weakly bound electrons play a key role inside CPs/GD composites.

## 1. Introduction

In the context of a higher energy demand assigned to an increased population and thus an increased level of needs, together with the depletion of natural resources and environmental pollution, the focus on finding alternative sources of energy (such as green or renewable energy) has also increased. Of all the eligible sources, e.g., the energy of the sun, wind, water, and thermal waters, the first is currently attracting the most interest. If it were efficiently converted, the energy from 1 h of sunlight on the entire globe would be enough to cover the need for one year of electricity [1]. Therefore, much research has been conducted to fabricate devices for converting solar energy into electricity and, as far as possible, to store it using the same device. Currently, the conversion process is made using silicon-based solar cells, and the trend is to replace these classical devices with lower-cost materials whose high conversion efficiency is similar. One of the newly tested devices is the DSSCs. They are conventional devices built from a photo-anode and a counter electrode (CE), overlapped in a sandwich configuration, with a thin layer of electrolyte that fills the space between them. The photo-anode consists of a transparent conductive layer (TCL), which could be Indium-tin-oxide (ITO) or Fluorine-doped Tin Oxide (FTO), on which a thin layer of TiO_2_ is deposited and dipped in a dye solution (usually N719). The outer part is represented by the CE, usually made of Pt, deposited on a transparent conductive layer (TCL). The electrolyte is represented by an I^−^/I_3_^−^ redox couple, mostly liquid.

**Figure 1:**
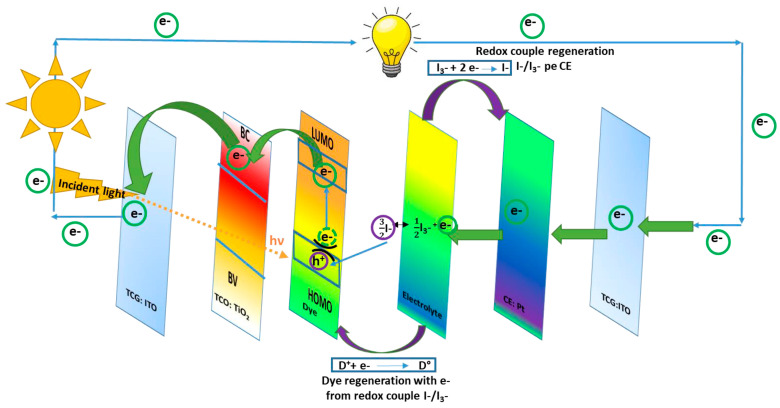
Mechanism inside a DSSC device.

DSSCs could also be bifacial, illuminated on both sides. They include a CE material with a double function, which works as a charge transfer agent and as a regenerator for the redox couple. The CE for this kind of DSSC is transparent, with illumination available from both the front and rear sides [2]. Of all DSSC components, the fundamental one used to convert the luminous energy into electric energy is represented by the photo-anode. The most common semiconductor used for this role inside DSSCs is TiO_2_. The mechanism inside DSSCs consists of the path followed by the solar light from TCL until the separated charge (namely the electrons) is loaded into the external circuit. When the radiation enters the transparent conductive layer (for example, TiO_2_) impregnated with dye, the radiation excites the dye molecules. Therefore, the dye molecules move to higher energy levels, namely the lowest unoccupied molecular orbital (LUMO), and from there, the electrons are promoted into the TiO_2_ conduction band (CB) and move across to the external electric circuit. When the electrons arrive at the conductive transparent electrode (TCE), they are collected and transferred to the CE, which is usually made of platinum (Pt). The dye molecules remain in an oxidized state after light exposure and regenerate by accepting an electron from the electrolyte redox couple. After that, they return to the fundamental state. This mechanism of the charge transfer inside the DSSCs device is shown in Figure 1.

Researchers who design and test DSSCs encounter many difficulties regarding sensitive issues about the way the components work inside the DSSCs. For example, the flexibility, long-term stability, active surface area (SA) and transparency of CE and TCL, the absorption efficiency of light, charge recombination, and so on. Amongst all of these, the main drawback of DSSCs remains the difficulty in controlling the charge recombination process, which is responsible for a major decrease in conversion efficiency. The last is the reason for being reported only on a few occasions: a conversion efficiency higher than 11% under diffuse daylight [3]. Excited dye molecules and other acceptor species from the electrolyte are involved in charge recombination processes, capturing electrons from the system and thus remaining unavailable to further interactions involved to complete the electric circuit. When using a flat surface of dye, less than 1% of incident monochromatic light is absorbed. One way of improving this performance is to increase the area of the active surface of TCL on which the dye is adsorbed, for example, by thermal sintering treatment of the TiO_2_ before depositing onto ITO or FTO substrates [4]. Moreover, dye molecules play an important role in the main process of the DSSC mechanism. Attached to the TiO_2_ surface, these absorb light, broadening the range of wavelengths to be absorbed. Then, the electrons are injected from the LUMO into the TiO_2_ conduction band. From this point, the electrons enter the semiconductor layer and enter the external circuit. At the same time, the oxidized dye molecules regenerate to the neutral state by reduction of the redox species in the electrolyte solution. Because it is a complete circuit, it runs without material consumption by generating electricity from sunlight. A substantial amount of this energy is unfortunately lost through the recombination process of electrons inside TiO_2_ with oxidized dye molecules or molecules in the electrolyte/redox medium. To increase the performance of the DSSCs, each component must be properly chosen. For example, the suitable sensitizer must fulfill several demands, such as: absorption in the full visible domain with the ability to use a higher percentage of light, affordable location of molecular orbitals (highest occupied molecular orbital (HOMO) and lowest unoccupied molecular orbital (LUMO)) to inject the electrons into the CB of the photo-anode and to help to regenerate the oxidized sensitizer from the redox electrolyte. In addition, the aggregation of the sensitizer molecules must be avoided by choosing a certain molecular structure and also the charge recombination from the TiO_2_/electrolyte interface. To improve the charge injection, functional groups such as carboxyl and phosphonate are desirable and the sensitizer should be also photo- and thermic-resistant providing long-term stability for the DSSCs device. Considering all of the listed demands, several types of sensitizers have been reported into the literature, including porphyrins, phtalocyanines, and metal-free organic dyes [5]. From all of these sensitizers, the biggest efficiency was reported on ruthenium and porphyrin dye with the advantage of availability and ease of structural tuning, possessing high extinction molecular coefficients [6,7]. Only a few of them reported power conversion efficiency (PCE) values higher than 9% when combining with iodide.

An issue being increasingly studied is the relationship between the electrolyte couple and the dye. It seems that the preferred redox electrolyte couple is I^−^/I_3_^−^ due to several characteristics such as good solubility, low absorption of light, appropriate redox potential (0.35 V) providing dye rapid regeneration; this couple poses a very slow kinetic of recombination between the electrons from TiO_2_ and the oxidized entity of the redox couple (I_3_^−^). They do not involve into the recombination reaction by contrast other sensitizers that bound I^−^ or I_3_^−^. Because the difference between the oxidation potential of the standard sensitizer (based on porphyrins and ruthenium, 1.1 V) and that of the redox couple (I^−^/I_3_^−^, 0.35 V), which means that the reduction potential of the oxidized dye is 0.75 V, this process provides the biggest potential lost from the DSSCs devices [8]. This value must be reduced at least to half of the value in order to increase the PCE to 15%. In order to move towards this, some aspects should be considered. From the regeneration of the oxidized sensitizer with I^−^, the reaction leads to the formation of the diiodide radical as a secondary product (I_2_^−^). Therefore, the redox potential of the I_2_^−^/I^−^ couple should be considered when determining the force of the sensitizer regeneration because I_2_^−^ leads to I_3_^−^ and I^−^ formation which is the main reason for decreasing the potential energy.

Another problem is the use of an expensive and rare CE material such as Pt, which implies the need to obtain Pt-free or low Pt content CE. Materials suitable for this position must be highly conductive, transparent, with a high rate of charge transfer, resistant to corrosion in electrolyte medium, low in cost, and available. Therefore, materials such as carbon and conducting polymers (CPs) structures or their combination thereof are eligible for this position [9]. Used alone, CPs do not perform very well as a CE inside DSSCs. In addition to their advantages, such as the conjugated structure, ease of preparation, availability and good stability, and possibility of depositing uniform thin film with a good adhesion on transparent conductive oxide (TCO), they have a relatively low conductivity. This drawback could be overcome by combining CPs with CNs for giving rise to composite materials with enhanced electrochemical and catalytic activity, able to ensure fast charge transfer from the external circuit to the electrolyte, supporting the regeneration process of the redox species. The reversible redox behavior of CPs is also a plus when it comes to contribution to a low charge transfer resistance (as in the case of polyaniline (PANI) and poly (3,4-ethylenedioxytiophene) (PEDOT)). As for the poor dispersibility of CNTs in common solvents and the tendency of GDs to overlap, leading to a graphite structure restoration, these problems are easily solved when CNTs and GDs are incorporated into the polymer matrix, so that charge transfer is accelerated and the specific active surface area is enlarged. Functionalized CNTs facilitate the formation of covalent bonds with the CPs through which the charge transfer occurs more easily and quickly; meanwhile, both GO and RGO, due to the functional groups on their surface, contribute to the uniform deposition of conductive polymers. Moreover, the charge transport is facilitated through π-π stacking between GD and CPs which creates multiple and shorter routes for both ion and electrons diffusion.

The first DSSC was made by depositing transparent layers of anatase on glass substrates, over which a thin layer of sensitizer, namely trimeric rhutenium complex, was dispersed. Using a sandwich configuration, an electrolyte thin layer containing the redox couple I^−^/I_3_^−^ filled the space between the Ru complex/TiO_2_/glass and the CE made of glass covered with monolayers of Pt. At a filling factor of 0.76, the efficiency conversion was 7.9% and raised to 12% under diffuse light exposure [4].

**Figure 2:**
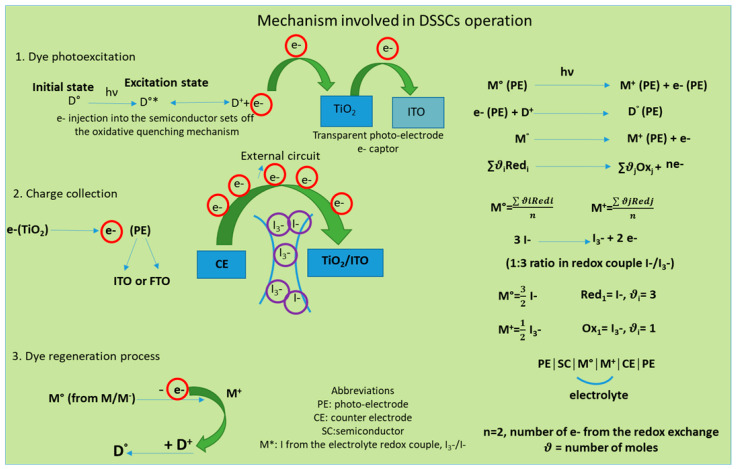
Mechanism of excitation and regeneration which underlines the DSSCs functionality.

From the entire mechanism of DSSCs, the materials used to design the CE are discussed here. The CE plays an important role, capturing the electrons from the external circuit and transporting them to the electrolyte where I_3_^−^ accepts electrons and moves to I^−^. To complete the electric circuit, following the dye regeneration pattern, I_3_^−^ accepts two electrons and changes to I^−^, which further regenerates through charge transfer from the CE during a reduction process (see Figure 2). The electron exchange involved in redox couple regeneration (electrons given up by I^−^) is fast enough to ensure efficient dye regeneration, while I_3_^−^ accepts electrons from the photoanode, and the process is slow enough to allow a high carrier collection efficiency.

The first materials tested as possible CEs were the carbon nanostructures such as graphene and CNTs, due to their high conductivity and low surface resistance, transparency, relatively fast charge transfer, availability, and affordability. Therefore, carbon nanostructures were used with this purpose from 1991 [10], when CNTs had been reported to improve the DSSCs performances, and this was followed by the use of graphene as a CE in DSSCs from 2013 [11,12,13]. In order to exceed the maxim PCE (7.88%) reached using carbon nanostructures [12], other materials were also tested, as for example their composites with CPs [13] or pristine CPs [14].

Combining CPs with CNTs, the perfect network is created and so the charge transfer takes place faster and more easily. Inside composites, different functionalizations, namely covalent or non-covalent, are formed between the functional groups attached on the surface of the CNT walls and the functional groups in the polymer backbone, which further provide the so-called synergistic effect.

## 2. Synthesis and Vibrational Properties of CPs-CNTs Composites

### 2.1. Synthesis of CPs-CNTs Composites

The main CPs which form the composites, used as CE, are poly(pyrrole) (PPy), PEDOT, and PANI. The CNTs, pristine [15] or trapped inside a gel [16], are the first carbon structures used as a CE material, replacing Pt. According to the literature, the composites based on CNTs and CPs were prepared by depositing CNTs on the substrates of the type FTO or ITO followed by: (i) chemical polymerization; (ii) electrochemical polymerization [16,17,18,19,20,21]; or other methods such as (iii) the precipitation of the already synthesized polymer [22] and (iv) the doctor blade method [23]. The one which provides good control of the thickness and uniformity of the grown layer is the electrochemical method that remains the most used of all. Before depositing the composite on a conductive glass substrate, certain preparations were necessary. Therefore, H. Li and colleagues treated an FTO substrate to improve its hydrophilicity by sonication into a mixture of ammonium hydroxide, water, and hydrogen peroxide (1:5:1 volume ratio) [24]. Then, on the already-prepared substrate, the CNTs were spin coated and then exposed to a heat treatment at 60 °C for 30 min. H. Li and co-workers have also reported preparation of PPy/CNTs composites using an in situ electro-polymerization technique. Prior to the preparation of the composite, the CNTs were treated to improve their solubility by functionalization with –COOH groups. This procedure was performed for improving the CNTs solubility in water by functionalization of the CNTs walls with –COOH functional groups [24]. Further, the composite based on CNTs and PPy was obtained from a mixture, containing 10 mM pyrrole, 20 mM sodium dodecyl sulphate (SDS), and 20 mM lithium perchlorate (LiClO_4_), through cyclic voltammetry (CV) [19,24]. Using the same method, namely the electrochemical synthesis, a composite with a honeycomb morphology was obtained [25]. To prepare PANI- and single-wall carbon nanotubes (SWCNTs)-based composites by electrochemical synthesis, Bumika, M. and colleagues used sodium dodecylbenzene sulfonate (SDBS) to obtain a CNTs dispersion of carboxyl-functionalized SWCNTs (SWCNTs-COOH) with a weight ratio of SDBS: SWCNTs–COOH equal to 9:2, in 0.5 M H_2_SO_4_ solution [20]. The resultant dispersion was then mixed with a second mixture prepared from ZnO (6 wt.%) and 0.25 M aniline (ANI) solution and deposited on FTO through CV, in a three-electrode configuration cell, between −0.62 V and +1.2 V [20].

The second most used method to synthesize composites is the chemical polymerization. X. Liu and co-workers reported this method for the synthesis of CE composites materials based on three different CPs precursors and CNTs [16]. The chosen monomers were polymerized in the presence of CNTs embedded in the polyacrylic gel matrix (PAA). The process of incorporating CNTs into gel was conducted according to a protocol reported by Li and co-workers [26,27]. According to this protocol, 15 mL of CNTs aqueous homogenous dispersion was mixed with 1 g of hexadecyl trimethyl ammonium bromide and stirred at 80 °C for 10 min. Afterwards, 10 g of acrylic acid (AA) and 0.005 g of N, N-methylenebisacrylamide were added and stirred until homogenized. When all components were very well mixed, the polymerization reaction was started with potassium peroxydisulfate (KPS) (0.08 g). The reaction was carried out for 2 h, under vigorous stirring at 80 °C. The final product was freeze-dried for 72 h. After synthesizing the CNTs-PAA gel, pieces of it were dipped into solution of monomers, of ANI, 3,4-ethylenedioxythophene (EDOT), and pyrrole (Py), for 24 h, at room temperature so that the monomers could swell inside the gel. After dispersion of the swollen monomers inside CNT gels in KPS solution (0.03 M), an internal polymerization process took place with the formation of the corresponding conducting polymers. The final products were poly (AA-co-CNTs-Py), poly (AA-co-CNTs-co-ANI), and poly (AA-co-CNTs-co-EDOT) gel. As a part of the DSSCs device, the as-prepared composite gels were soaked into a liquid electrolyte containing tetrabutylammonium iodide, tetramethylammonium iodide, I_2_, tetraethylammonium iodide, LiI, and tetrabuthylammonium iodide in N-methyl-2-pyrrolidone and acetonitrile (1:4 volume percentage) [16].

Other methods, rather physical methods, used to prepare a composite based on CPs and CNTs were reported by Abdul Almohsin, S.M. and colleagues [22] and respectively by Dowa, C. et al. [23], using the precipitation of CPs on top of CNTs/FTO and, respectively, the doctor blade method.

According to the last method, PANI and CNTs were dispersed in m-cresol at a 100 mg/mL concentration until a viscous paste was obtained. During mixing PANI and CNTs, a few drops of terpineol and ethyl cellulose (15 wt.% in ethanol) were added and the whole mixture was magnetically stirred for 3 h. The resultant paste was spread over an FTO-coated substrate by the doctor blade coating method. The coated layer was maintained at room temperature to dry and then thermally treated at 400 °C for half an hour. M-cresol interacts with the polymer chains and act as a dopant to PANI and, at the same time, it is a good dispersing agent for high CNT content. Mixing CNTs with m-cresol produces a thick, viscous solution that helps to deposit a more uniform and homogenous layer of CNTs on a substrate.

Composite materials used as CE after being deposited on FTO substrate show different features depending on the type of CPs, the carbon nanostructures inside the composite, the interaction between the two components of which is directly related to the type of synthesis. Moreover, when using carbon nanostructured inside the composites, there are some aspects regarding their functionalization to be eligible for further interaction with the CPs. Then, considering all these aspects, the vibrational properties will be further discussed, investigated using mainly Raman and FTIR spectroscopy and some parameters concerning the DSSCs electro-catalytic activity. Certain aspects will be followed in order to understand the interaction between the composite components and its influence on the light conversion efficiency, according to the information provided by the selected papers used in this work.

### 2.2. Vibrational Properties of CPs/CNTs

**Figure 3:**
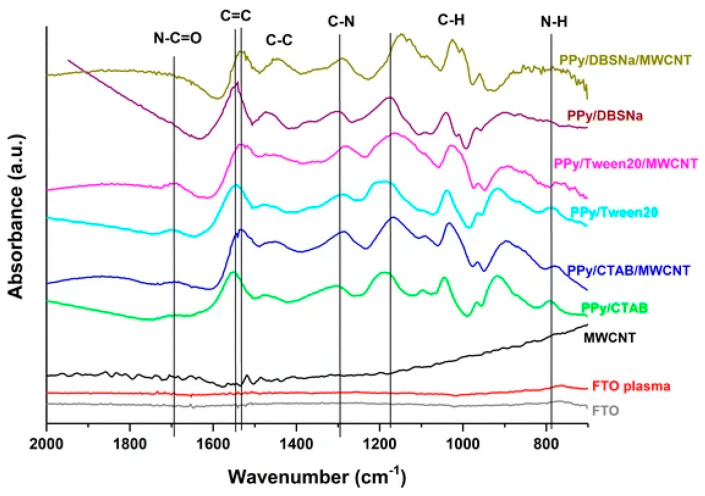
FTIR spectra of FTO, FTO plasma, MWCNT, PPy/CTAB, PPy/CTAB/MWCNT, PPy/Tween20, PPy/Tween20/MWCNT PPy/DBSNa, and PPy/DBSNa/MWCNT, where MWCNTs are non-covalently functionalized with PPy [17].

Prepared by in situ chemical polymerization of PAA–CNT gel soaked into monomer solution, poly (AA-co-CNTs-Py) was analyzed through IR spectroscopy and the recorded spectra revealed specific absorption bands assigned to Py, CNT, and PAA. According to Table 1 (inserted below) the specific bands were assigned to PPy ring vibrations (1547 and 1038 cm^−1^), N-H in-plane deformation [28,29], N-C stretching vibrations, and C-H band stretching vibrations (1175 and 910 cm^−1^), respectively. Meanwhile the band situated at 3421 cm^−1^ corresponds to the stretching vibration of the OH functional group of the PAA polymer, while the bands assigned to CNT are those located at 1450, 620, 1400, and 2945 cm^−1^ corresponding to wagging vibration, C-H out-of-plane vibration, the last two being characteristic of the methyl functional group and sp^3^ hybridized carbon atom. Other bands are assigned to the external functional group attached to the CNTs walls, such as for example: for the carboxyl group, specifically the bending vibration of C=O bond in –COOH represented by the IR band at 1685 cm^−1^, the C-O-C stretching vibration and the assigned band at 1140 cm^−1^ [30,31], the C=O stretching vibration and the bending vibration of the –OH bond in –COOH at 1719 and 1348 cm^−1^. For a better understanding of all interactions between PPy and grafted CNTs with carboxyl groups, the IR vibrational structure of this composite is shown in Figure 3.

According to X. Liu et al., gel electrolytes with CNTs incorporated have a dual function inside DSSCs: to enlarge the SA, as well as increase conductivity, providing a high catalytic activity and thus contributing to short-circuit density (Jsc) enhancement [16].

Changes in the IR absorption bands of PPy have been reported in the case of the IR spectra of the composite based on PPy and CNTs, synthesized through the electropolymerization of Py in the presence of CNTs [17]. A shift to lower wavenumbers of the band located at 1530–1560 cm^−1^ and assigned to the C=C/C-C stretching vibration of the PPy chains indicates a higher delocalization length in the polyconjugated system [32], which means longer polymeric chains are formed in the presence of CNTs, because of a non-covalent interaction between the π-π bonds of PPy and CNT (see Figure 3).

The presence of functionalized multi-wall carbon nanotubes (MWCNTs) into the PPy matrix is revealed through an enhanced intensity of peaks and a little shift, as a consequence of the interaction between carboxyl group from MWCNTs and functional groups from PPy creating a network where electrons are transferred from one compound to the other. Further, inside the nanocomposite based on PPy and functionalized multi walled CNTs (FMWCNTs), the bond associated with C-H, C-C, and N-H vibration becomes weaker and instead the C-N bond becomes stronger. The fact that MWCNTs are wrapped in PPy is confirmed through the disappearance of IR bands, such as for example the IR bands located at 1198 and 2879 cm^−1^, present in acid-treated MWCNTs [33,34]. As formed PPy-FMWCNTs have a better conductivity than pristine PPy, 250 S/cm vs. 35 S/cm due to a higher localization length of 10 nm vs. 1.55 nm for FMWCNTs, the value improved as a result of the large arrangement of the π conjugated structure [21].

The electrostatic interaction which takes place between the quinoid rings of both components, one donor (PPy) and the other e^−^ acceptor (MWCNTs), determined a fast movement of charges inside the composite enhancing its conductivity. Morphologically, the PPy layer is porous and uniformly dispersed on the electrode surface, and embedded on FMWCNTs which creates a transport network for electrons that enhances the cathodic reaction of the redox couple I_3_^−^/I^−^ [35].

**Figure 4:**
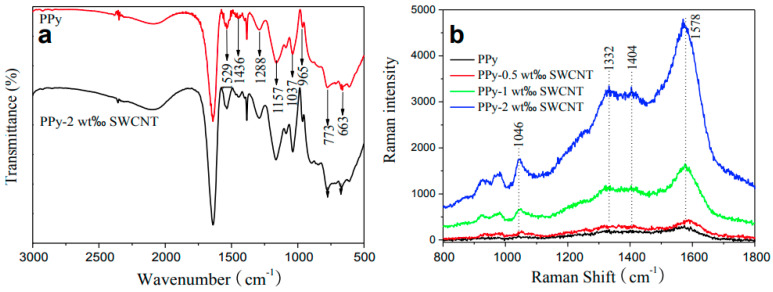
(**a**) FTIR and (**b**) Raman spectra of PPy and PPy-SWCNT composites, where SWCNTs are covalently functionalized with PPy [36].

According to He and co-workers, using in situ chemical polymerization, CNTs are covalently bond to PPy, forming a new composite [36] (Figure 4) with a covalent bond between the nitrogen atom in the pyrrole ring and the sp^2^-hybridized carbon atom in the CNT network. The best results have been recorded for an optimum of 2 wt.% SWCNTs on which values of 8.3 PCE have been reported. Compared to pristine PPy (PCE 6.3%), the improved results were assigned to the lower charge-transfer resistance (Rct) value.

In all Raman spectra of the PANI-CNTs composite, the CNT-specific lines were located at 1591 and 1334 cm^−1^ corresponding to the radial breathing mode (RBM) [37] and tangential mode [38] and the PANI lines located at 1581, 1052, 1083, 1330, 1370 cm^−1^ assigned to the vibrational modes of C=C stretching, C-H in plane deformation, and the aromatic ring stretching mode [39,40] (see Table 1 for more information). The most significant change has been recorded in the intensity of the Raman line located at 1334 cm^−1^ which increases with the amount of SWCNTs in the composite. The interaction between the components inside the composite was reported to be rather weak considering the lowest PL intensity band located at 520 nm [16]. The layer morphology, noted by PANI and CNT-based composites, varied from the honeycomb structure [24] to the axel sleeve structure obtained by co-polymerization [41] or a uniform film obtained by electrophoresis and CV [42].

It was already reported that the deposition of a PEDOT thin layer on the TCO substrate contributes significantly to a decrease in the surface resistance (SR) and as a direct consequence the conductivity increases. When PEDOT is prepared by the electropolymerization of EDOT, a layer of well-connected mesoporous composite is obtained on the CNTs film already cast on the TCO substrate. In this case, the CNTs play the role of template leading to porous nanostructured wires [43] but the CNTs can also be wrapped in PEDOT film by π-π coupling [44]. If another method is used, such as in situ chemical polymerization, covalent bonds are formed between PANI and CNTs, more precisely between –NH– in PANI and –C=, i.e., the sp^2^ hybridized carbon atom of SWCNTs, and this bond contributes significantly to the acceleration of charge transfer between composite components [45]. The CE improves with increasing CNTs loading, regardless of the chosen synthesis method [46].

Composites based on PEDOT and CNT have been reported having different morphologies, such as porous wire nanostructures deposited on a CNT template [43], or core-shells structures, where CNT is the core and PEDOT is the shell [47], using the oxidative polymerization method. It was found that, when using an aligned structure or well-ordered CNTs, it could enhance photovoltaic performance, i.e., PCE, by decreasing resistance and increasing conductivity [15]. Different from the classical CNT and CP–based composite, CNTs could be also used as a filler or matrix with different CPs. The latter has been used to solve both the problem of the poor dispersion of CNTs in solvents and to improve their electronic conductivity. Another study reported as an electromaterial (EM) in DSSCs a poly(3,4-ethylenedioxythiophene)-poly(styrenesulfonate) (PEDOT: PSS)-based composite and CNTs, where PEDOT: PSS was used as a dispersing agent of CNTs [48]. The efficiency of the composites thus prepared was lower than those reported when Pt was used as a CE (8.5%).

**Figure 5:**
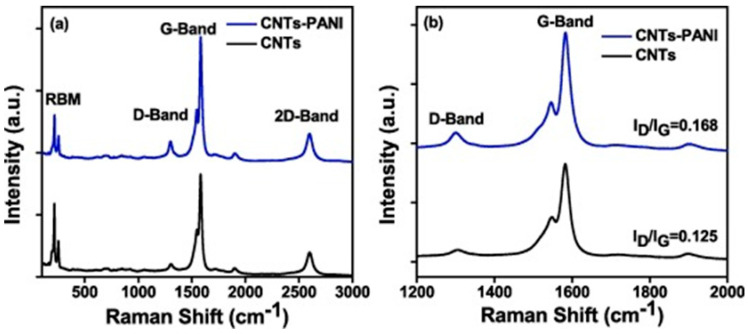
Raman spectra of (**a**) CNTs, CNTs-PANI films coated on FTO glass, and (**b**) a magnified version of the D and G band [23].

Inside PANI and CNT-based composites, the resultant defects generate a higher surface area, and this is supported by an enhanced intensity of the D band peak in the Raman spectrum of the composite compared with the spectrum of the CNTs, which confirms the presence of sp^3^-hybridized carbon atoms attached to the surface of CNTs. The value of the I_D_/I_G_ ratio increases from 0.125 to 0.168 for the composite (see Figure 5) due to the increase in the number of defects at the edge of the CNTs [23].

**Table 1:** Main specific IR and Raman bands assigned to the components used in the synthesis of CE composite materials (PPy, PANI, PEDOT, PAA, and CNTs).

CPs	Vibrational Modes Active in IR Spectroscopy	Wavenumber (cm^−1^)	Ref.	Vibrational Modes Active in Raman Spectroscopy	Wavenumber (cm^−1^)	Ref.
PPy	Vibration of pyrrole ring ν C–H ν N–C δs N–H (1038 and 1547 cm^−1^) ν C=C and ν C–C, PPy ring vibrations	700–800 910 1175, 1210 1038 1547 1530–1560 1556	[16,28,29,32]	-	-	-
PANI	δ C–H of the quinoid ring ν C–N and δ C=C ν quinoid ring and δ benzoid ring	1133 1243 1301 1489 1564	[40,49,50,51]	Bipolaron and polaron bands, δ C-H, ν ring, ν C=C	940 990 1052 1083 1330 1334 1370 1581	[18,39]
PEDOT	ν C−C or C=C of the quinoide structure and ν thiophene ring	834 978 1187 1315 1356 1513	[52]	ν C-S-C bond in thiophene ring δ C-O-C bending vibration in ethylenedioxy group ν_as_ SO_2_	834 978 1187 1315	[52]
PAA	ν OH	3421	[16]	-	-	-
CNTs	γ C–H	620 1140 1348 1400 1450 1685 1719 2945	[16]	RBM, E_2g_ mode assigned to slightly disturbed graphite E_2g_ mode of graphite wall	1334 1591	[16]
	ν C-O-C					
	δ OH from –COOH					
	ω C–H (1400 and 1450 cm^−1^)					
	δ C=O from –COOH (grafted to the CNTs wall)					
	ν C=O					
	ν CH_3_					

### 2.3. Performance of CPs/CNTs as CE in DSSCs

The efficiency of the presented composites varies from 1.67% [17], reported on PPy/CNTs, to 9.07% for PEDOT/CNTs [53], both composites synthesized through in-situ electropolymerization of monomers (see Table 2). Higher PCE values were obtained on composites when the dispersing agent (SDS) was used, the PCE value of 6.15% being close to that reported for Pt (6.36%) [20]. The good results were mainly attributed to the low Rct at the CE/electrolyte interface [18]. Also, very good results have been reported for structures obtained by chemical vapor deposition (CVD) of the polymer on the CNT film surface. Such an example is the subject of the study by W. Hou and co-workers, when the presence of PPy induced a PCE of 7.15 [54]. Comparing a covalent composite [36] with those in which the interaction between components is non-covalent [17], it would appear that those with a higher PCE are those in which covalent bonds are present (see Table 2).

For describing the electro-catalytic activity of the CE, there are several important parameters and associated measurements which should be considered, namely CV, electrochemical impedance spectroscopy (EIS), Tafel polarization, chrono-amperometric studies, R_CT_, surface layer resistance (Rs), PCE, Jsc, filler factor (FF), and so on. The information discussed in this section depends on the information provided by the papers selected for this study.

In addition, the photovoltaic performance of DSSCs is investigated though I-V measurements. EIS studies are performed to describe the charge transfer kinetic of the electrochemical system, more precisely the electro-catalytic activity of the CE versus the reduction process of the electrolyte, in a symmetric cell system. Using the Nyquist plot, the R_CT_ could be determined from the graphic which describes the charge mobility between the CE and the electrolyte. Specifically, the R_CT_ value depends on the diameter of the semicircle corresponding to the chemical capacitance (CPE) at the electrode/electrolyte interface. The semicircle on the right, located in the low frequencies area, represents the Nernst diffusion impedance (W), a measure of the electrolyte that controls the diffusion of the I_3_^−^/I^−^ redox species to the CE, in addition a lower value of R_CT_ indicating a faster charge transfer inside the symmetric cell. R_CT_ varies inversely with the Jsc recorded for I_3_^−^ to I^−^ reduction process on CE. From the cyclic voltammograms recorded using electrodes of the type PEDOT/ITO, CNTs/ITO, ITO, and PEDOT-SWCNTs/ITO, the synergic effect of both components is presented. While on the pristine ITO electrode the redox peaks are missing, in the case of PEDOT/ITO, there are two peaks that are broad and far apart, and less reversible than those found on PEDOT-SWCNTs/ITO, revealing the idea that PEDOT alone could not be as efficient and that this could be extended to replacing the ITO substrate with SWCNTs as a better one for improved catalytic response. Another evidence that PEDOT and SWCNTS go well together is the efficiency value which is double in the case of PEDOT-SWCNTs than for pristine SWCNTs. A high filling factor and I-V measurement confirm the good catalytic performance of PEDOT-SWCNTs composites used as a CE for DSSCs devices [55].

According to Abdul Almohsin, S.M., the composites based on PANI and SWCNTs, prepared by one-pot electrochemical synthesis [22], present a much lower R_CT_ value than the pristine PANI (95 vs. 845 Ω), proving the contribution of the SWCNTs to the enhancement of charge transfer within the composite. The morphology of the CE films also contributes to the photoelectric performance of the DSSCs by influencing their transmittance and diffuse reflectance properties. The latter two are maintained at high values even when CNTs have been added [53]. The high conductivity of SWCNTs together with the layer morphology [24,53] doubled the photoelectric performances of the CE in DSSCs. This is the case of composites based on PPy and CNTs with a honeycomb-like structure [20]. The presence of a surfactant within the EM composite can influence its catalytic performance [17,19]. The surfactant induces an enhancement of the interactions between the polymer chains, increases the stability, and enhances the electropolymerization current, together with providing an easy charge exchange between the electrolytic medium and the polymers [56].

Benefiting from a morphology of the porous structure resulting from well-separated fibers formed from PANI-coated CNTs, as a result of the doctor blade synthesis method, the PCE value of CNTs-PANI (6.67%) almost reaches the value reported for Pt (7.70%) [23]. The low performance of this CE EM could be due to the thick catalytic layer with 6% PANI content, which provides a higher total internal resistance, but also the opaque nature that prevents the light reflection effect [43].

In terms of DSSCs performance, the external quantum efficiency (EQE), also called the incident monochromatic photon-to-electron conversion efficiency (IPCE), is an essential characterization method.

**Figure 6:**
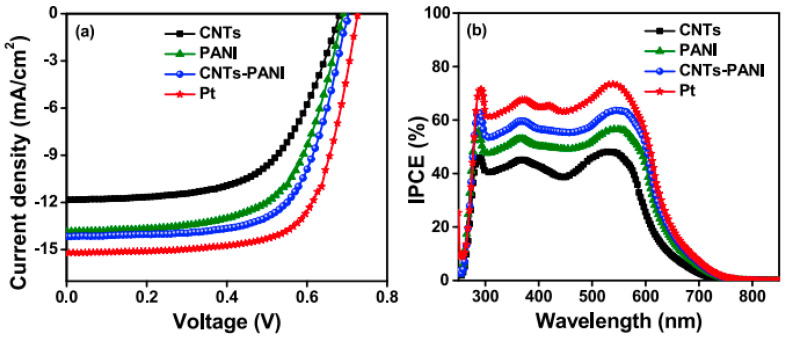
(**a**) JV characteristics of Ems, namely CNTs, PANI, CNTs-PANI, and Pt and (**b**) the incident photon to current conversion efficiency (IPCE) spectra of CNTs and CNTs-PANI-based DSSCs for 10 devices [23].

IPCE spectra showed a broad band in the 250–800 nm region, with a maximum value at 535 nm. From Figure 6b, it can be seen that the IPCE curves of DSSCs increase as a function of EM in the order CNTs < CNTs-PANI < Pt. The reported results obtained in the IPCE curves are well correlated with the JV curves (Figure 6a). Despite the fact that the performance of CNTs-PANI CE materials does not exceed that of the Pt electrode, CE composites based on CNTs and CPs could be a good alternative to replace Pt, at least economically.

## 3. Synthesis and Vibrational Properties of CPs-GD Composites

### 3.1. Synthesis of CPs-GD Composites

After the incorporation of graphene into PEDOT, the electrochemical activity is reported as improved and the charge transport is much faster through the composite film [57]. The reduction potential of the redox couple is shifted towards negative potentials, compared to a Pt/ITO CE, which means it still has a higher resistance. When prepared by CVD, the characteristic parameters of the graphene layer are superior to those prepared using other techniques. For example, the I_2D_/I_G_ ratio is about 2.5 while the D band has a low intensity and the transparency of the monolayer compared to four layers is of 97.4% vs. 90.6%, at λ = 550 nm.

Moving from mechanically mixing graphene [58,59] and CP to the deposition of a polymeric film on top of the graphene, ultimately by in situ polymerization [60,61], many methods of synthesizing composite that were designed as CE materials have been reported. Together with the organic sol-gel route for the synthesis of aerogel structures [62], all reported methods have in common the individual deposition of the polymer and the graphene/GD layer; this means that the resulting composite material is closer to a sandwich structure more than to a bulk one for which the polymerization process takes place simultaneously [63,64,65].

For the PANI/graphene composite, the reaction mixture was prepared by combining a solution of monomer, prepared from 1.2 mmoles of ANI dissolved in 50 mL acid solution (0.4 M HCl) with 50 mL of a second solution made by solubilizing 0.41 mmol ammonium persulphate (APS), as a polymerization initiator, in 0.4 M HCl. The mix of both solutions was poured into a previously cooled bath, into which the graphene-modified electrode was already been introduced, and left there for 30 min, and then immersed in a 1 M HCl solution until complete conversion occurred from polyaniline-emeraldine base (PANI-EB) to polyaniline-emeraldine salt (PANI-ES) [66]. Brought together, the pristine graphene, difficult to incorporate into a polymer matrix, and the PANI difficult to adhere to the FTO substrate also came with advantages such as providing appropriate support for nucleation and polymerization, due to the presence of GO, and the homogenous RGO dispersion provided by PANI, making them a winning combination for a CE material. At the interface of both PANI and RGO, interactions such as π-stacking, hydrogen bonding, electrostatic, and donor–acceptor take place [67]. In an acidic medium, between PANI, in the form of emeraldine salt (characterized by a polaron energetic state) and RGO with a negative charge storage capacity, a transfer of weakly bound electrons takes place, which is a good source of electrons for the regenerating of the electrolytic redox couple I^−^/I_3_^−^.

A special method for preparing aerogels composite structure was reported recently by Mohan, K. et al. The conversion into a PANI/RGO aerogel (PANI/RGOA) composite was made by preparing two dispersions of GO and, respectively, PANI nanotube (PANI NT), in deionized water, of 10 mg/mL in each suspension. In the preparation of RGOA, GO aqueous suspension was mixed with resorcinol, formaldehyde, and sodium carbonate (0.337, 0.362 g and, respectively, 1.6 mg) and stirred for 30 min. The two suspensions were then mixed in 100 mL flasks, sealed, and maintained at 85 °C for 3 days to obtain PANI NT/RGO aerogels, washed with deionized water, and freeze-dried for 24 h. At the end of the process, the aerogel pastes were deposited through the doctor blade method onto FTO glass and heated to 80 °C to obtain the CE. The aerogel pastes contained 10 mg of PANI/RGO aerogel dispersed in 0.5 mL Nafion solution [62]. The PANI/RGO aerogel prepared via the organic sol-gel route presented a high surface area and excellent catalytic activity towards the reduction of I^−^/I_3_^−^ in electrolyte [62]. PANI/GD composites have been also synthesized using economical methods such as the spray method [68]. First, the RGO layer was deposited on the FTO surface from 0.015 g of RGO mixed with 2.5 mL of acetic acid and 0.02 Triton X-100, and finally 100 mL of ethanol was added, and the entire mixture was ultrasonicated for 1 h. The resulted mixture was sprayed on the FTO surface, thermally annealed at 100 °C, and finally sintered at 250 °C. Then, 0.015 g RGO powder was mixed with an appropriate amount of PANI, 2.5 mL acetic acid, and binding agent Triton X-100, for 10 min; after that 1 mL dispersion of SnO_2_ in ethanol was added, followed by pouring 100 mL of ethanol. The entire mixture was then sonicated for 1 h and at the end, PANI/RGO/SnO_2_ was obtained. The suspension was sprayed onto a heated FTO support and sintered at 250 °C. The resulting composite has a porous structure, with many interconnections and pathways between components that provide an enhanced electrocatalytic effect in the triiodide ion (I_3_^−^) reduction reaction at the CE. The porosity of the composite increase when SnO_2_ was introduced into the RGO structure provided a better adhesion for the interaction with CP. Comparing the PANI/graphene composite with the PPy/graphene composite, the way of synthesizing is very similar, so the composites materials were obtained by mixing the already-prepared polymer with the graphene precursor. Therefore, PPy was first obtained from mixing the aqueous solution of polyvinyl alcohol (PVOH) with ferric chloride, followed by the addition of pyrrole monomer, and the mixture was stirred in an ice bath for 4 h to obtain powder in suspension. The resulting powder was then mixed with graphene oxide (GO) powder in 10 mL of water. To this mixture, 0.088 g ascorbic acid was added, with the task of playing two roles, binder and reducing agent. The suspension was then deposited onto the FTO substrate using the doctor blade method [61]. In addition, the mechanical mixing method is not to be neglected in the preparation of PEDOT: PSS and graphene-based composites [58,59]. Using ultrasonication to mix PEDOT: PSS with nanoporous RGO helped to exfoliate RGO and insert a thin layer of graphene into the PEDOT: PSS matrix [59].

### 3.2. Vibrational Properties of CPs-GD

Regarding the vibrational properties of CP and graphene-based composites, leaving aside the characteristics of the IR and Raman spectra signature of each PANI, PPy, PEDOT, PEDOT: PSS, graphene, GO, and RGO, which will be also mentioned here in Table 3 inserted below, some changes were observed in their composite spectra that are relevant to be discussed here in terms of the type of functionalization between the two components inside the composites.

For further clarification, each individual polymer was discussed as follows: in the case of PANI, the active conductive form of PANI is ES, and after preparation and conversion it is important to check the presence of PANI-ES. Therefore, the conversion of PANI-EB to PANI-ES is confirmed by the presence of N in a protonated state. The distribution of N states shows the existence of pyridinic and pyrolic nitrogen [69].

**Figure 7:**
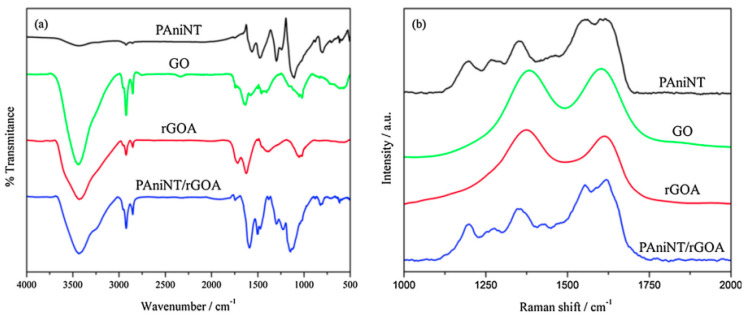
FTIR (**a**) and Raman (**b**) spectra of PANI NT, GO, RGOA, and PANI NT/RGOA [62].

Further evidence for the conversion from PANI-EB to PANI-ES is represented by the presence of the absorption band located at 330 nm, assigned to the π-π* electronic transition of the benzoid ring, which is blue-shifted from 330 nm (for bulk PANI) to 313 nm, a minor peak at 347 nm corresponding to π-π* electronic transition of PANI-EB, and the split peak from 360 nm assigned to the localized polaron-π* transition from the conductive form of PANI, the emeraldine salt. Another two bands located at 366 nm and 434 nm, corresponding to localized polarons and π-polaron transition, also confirm the formation of PANI-ES [70]. In addition, the main band characteristic of the insulating form of PANI-EB, located at 630 nm, assigned to local charge transfer between the quinoid ring and the adjacent imine-phenyl-amine is absent [71]. Further, for the PANI/RGO aerogel composite, both the Raman and FTIR spectra reveal the presence of both PANI and RGO [62] (see Figure 7), the bands from the spectra being overlapped, and the main change in their profile when analyzing the PANI/RGO spectrum seems to be the change in the main bands profile, described by an enhancement in their intensity rather than the appearance of new bands. The aerogel composite prepared by introducing PANI in GO suspension also presents new bands in the FTIR absorption spectrum, located at 3421, 1304, 1113, and 812 cm^−1^, assigned to stretching vibration of N-H bond, stretching vibration of C-N bond, stretching vibration of N-Q-N, and asymmetric stretching vibration of 1,4 double replaced benzoid ring.

The conversion of GO into RGO during synthesis was confirmed by the disappearance of the bands associated with the groups with oxygen and a decrease in intensity of the bands located at 3440, 1736, and 1402 cm^−1^, as revealed from the PANI/RGO FTIR spectrum. Complementary to the FTIR spectrum, the Raman spectra reveal the bands specific to PANI, located at 1620, 1550, 1350, and 1200 cm^−1^, associated with C-C vibration, imine vibration C=N, semi-quinoid polaronic vibration C-N^+^, and the in-plane bending vibration of the C-H bond corresponding to the quinoid ring, respectively [72]. The bands specific to GO and RGO, the G and D bands, are located at 1608 and 1360–1385 cm^−1^, respectively. H. Mohan and co-workers reported an enhancement of the I_G_/I_D_ ratio during aerogel formation, namely from 0.98 to 1.5, due to the conversion of GO to RGO associated with a decrease in the number of the oxygen groups from the surface, which also leads to a retrained sp^2^ domain surface. Nevertheless, the vibrational spectrum of PANI/RGOA does not differ much from the PANI spectrum, the main change consists of a decrease in intensity of the band located at 1620 cm^−1^, assigned to the presence of RGOA inside the composite structure. From the morphology point of view, the tubular fiber structure of PANI is weaving with the wrinkled paper appearance of the graphene compound, where there in not graphene or PANI. PANI is attached at the graphene foil surface without aggregation. Inside the aerogel structure, PANI interacts with RGO through π-π stacking at the basal plane level.

**Figure 8:**
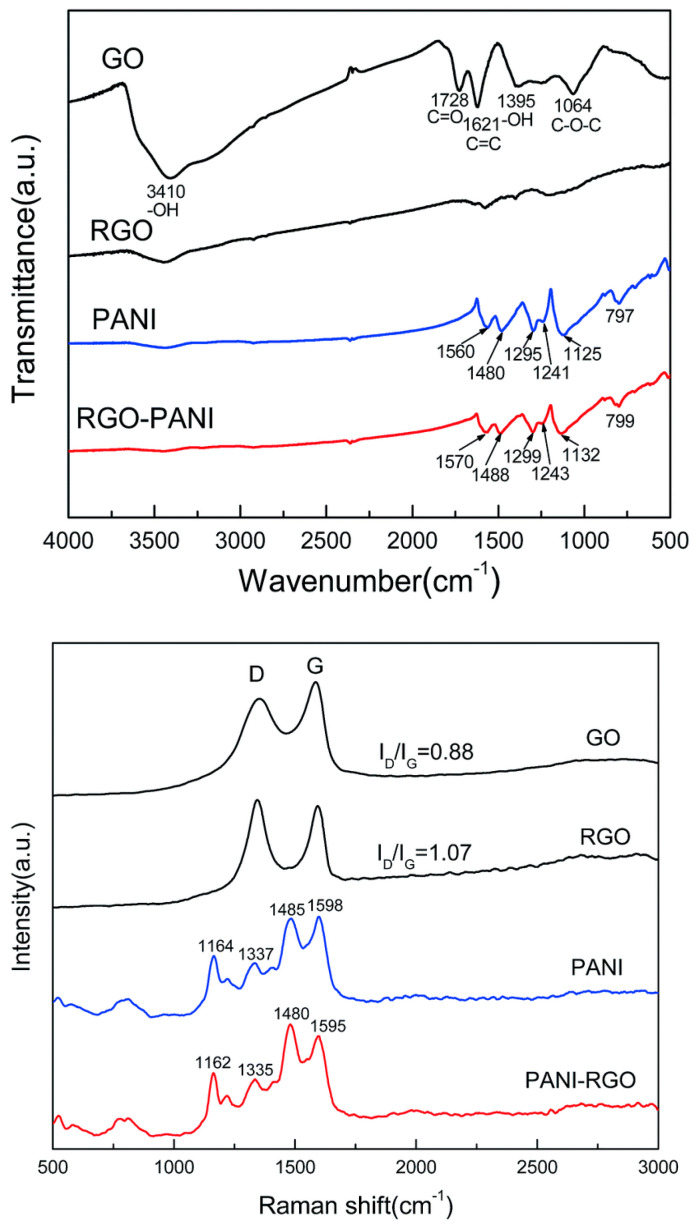
FT-IR and Raman spectra of GO, RGO, PANI, and PANI-RGO nanocomposites [73].

The non-covalent interaction between PANI and RGO, exemplified according to Figure 8, is described with the help of the red shift of the IR band, related to the PANI matrix, located at 1560, 1480, 1295, 1241, 1125, and 797 cm^−1^, as a consequence of a π-π interaction and hydrogen bonding between the basal planes of RGO and the PANI backbone [73]. Due to the strong interaction of RGO hydrophobic planes and PANI backbone [73,74], the Raman lines of RGO/PANI are shifted towards low wavenumbers. The peaks corresponding to the PANI signature have been red-shifted due to π-π interaction and hydrogen bonding between the basal planes of RGO and the PANI backbone [73], describing the non-covalent interaction between the components (PANI-RGO); a shift towards low wavenumbers has been also observed in the Raman spectra of RGO/SnO_2_/PANI as a consequence of the strong interaction of RGO hydrophobic planes and the PANI backbone [73,74].

When analyzing the composite based on PPy and GD, it seems that the CP is the prevalent component of the composite as in the cases of the PANI-based composite described above. Thus, the signal of PPy prevails on the spectrum of the composite [61]: in the Raman spectra of the composite with RGO, two main bands were revealed, located at 1341 and 1559 cm^−1^ and assigned to the stretching vibrations of the pyrrole ring and C=C bond from Py, respectively. When added to RGO, the main PPy bands shifts to 1350 and 1590 cm^−1^ upon increasing the amount of RGO as a consequence of the overlaps with the D and G bands of RGO [75]. Comparing the aerogel carbonic structures, the polymer gel with the classic shape of composite used as a CE material and electrolytes for DSSCs, the first attract more interest. For example, from the carbonic aerogel category, graphene aerogels have a mesoporous three-dimensional structure due to the interconnected graphene sheets, a structure which provide special properties such as a high electric conductivity [76] and large volume of the pores. These features facilitate the charge transport and the mass transfer of the redox species. Therefore, their special structure recommends them as appropriate substitutes for Pt CEs. Very good results have been reported when aerogels based on carbon structures have replaced CEs in DSSCs [77]. An increase in PCE from 7.07% [77] to 8.83% was reported [78].

Porosity could be successfully enhanced through other ways as for example by metallic oxide particles inside the CE material (carbonic structure, GD, RGO). The increased porosity of the resultant composite provides better adhesion for the interaction with CP and as a result, efficiency improved considerably (from 4.7% to 6.25%) when introducing oxide metallic NPs inside the RGO matrix. For example, SnO_2_ NPs increase the electrical conductivity [79] through contribution to the relaxation process of charge carriers [80]. All of these NPs are additional catalytic sites at the CE surface which are conducting and enhance the active surface area improving the DSSCs performances. Another aspect which could improve the PCE is the treatment of the photo-anode, containing two layers of TiO_2_ on the FTO, with TiCl_4_ (8.68%) [68]. The treatment was performed by dipping the photo-anode in a 0.04 M TiCl_4_ solution, at 70 °C, for 30 min, and sintering at 450 °C for 45 min [68].

**Table 3:** Synthesis and CE performance parameters (CE, FF, J_sc_, and R_CT_) of CPs/GD composites.

CPs/Graphene Composite	Synthesis	CE (%)	FF	J_sc_ (mA/cm^2^)	Rct (Ω)	Ref.
PANI/graphene	In situ chemical polymerization	3.58	0.473	10.683	0.346	[81]
PANI/graphene	In situ chemical polymerization	7.45	62.23	15.504	-	[60]
PANI/RGO aerogel	Organic sol-gel route	5.47	0.59	11.5	14.36	[62]
RGO/SnO_2_ NPs/PANI	Spray method	8.68	63	18.6	23.5	[68]
PPy/RGO	Chemical polymerization	0.05%	0.28	0.4	-	[61]
Graphene-Si_3_N_4_/PEDOT: PSS	Mechanically mixture	5.24%	0.71	10.16	49.13	[58]
PEDOT: PSS-PG	Ultrasonication	9.57%	16	76	0.92	[59]

### 3.3. Performance of CPs/GD as CE in DSSCs

**Figure 9:**
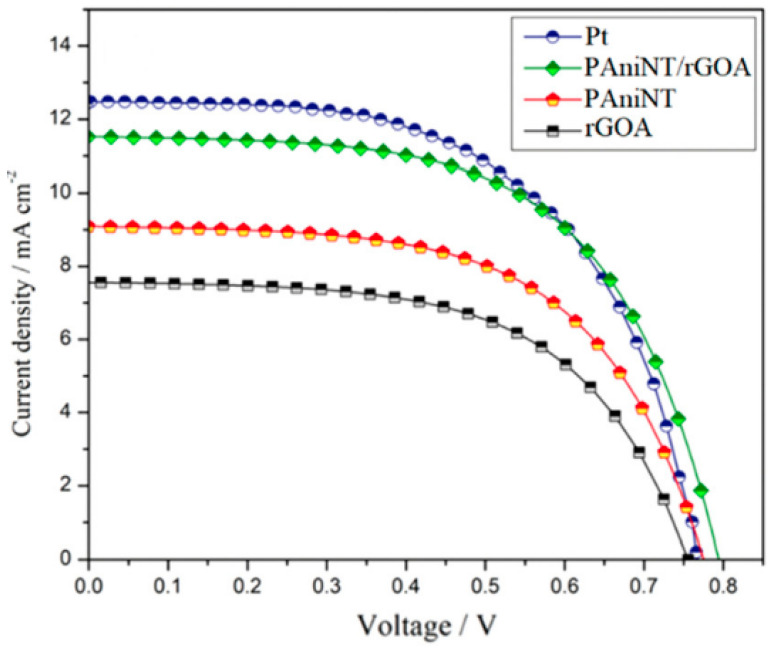

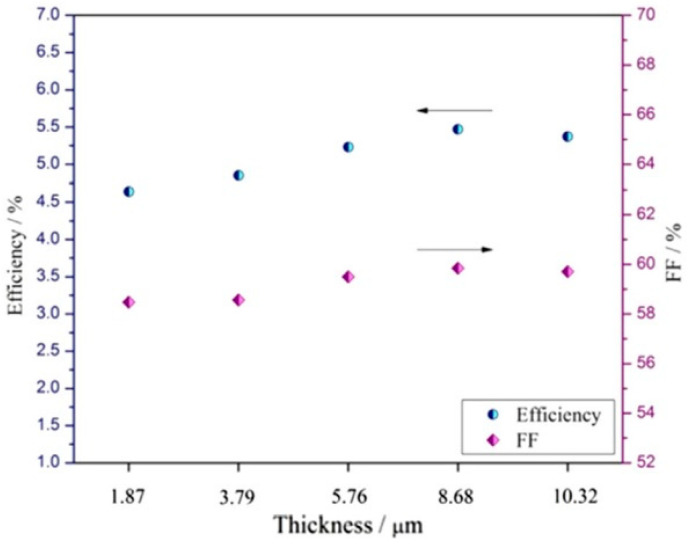
J-V characteristics of DSSCs fabricated with PAniNT, RGOA, PAniNT/rGOA, and Pt counter electrode (**top figure**) and photovoltaic parameters of DSSCs fabricated with PAniNT/rGOA CEs of different thicknesses under irradiation of 100 mW cm^−2^ light (**bottom figure**) [62].

The electro-catalytic properties of the CE materials inside the DSSCs are evaluated through EIS, CV, I-V, and Taffel polarization curves. From all these measurements, parameters such as R_CT_, RS, FF, J_sc_, and J_0_ are obtained, which are key factors that fully describe the CE and DSSCs performances, respectively. For all the parameters mentioned above, there is a short description at the CNTs/PCs section. Additional factors, e.g., the thickness of the CE layer, are also very important when considering the catalytic activity of a CE composite. The catalytic activity is directly related to the reduction process rate at the CE surface (I_3_^−^/I^−^) and the number of the catalytic active sites. The lowest efficiency was reported for DSSCs fabricated with the RGOA counter electrode (3.29%) with an open circuit-voltage (VOC) of 755.81 mV, short-circuit current density (J_SC_) of 7.59 mA cm^−2^, and a fill factor (FF) of 57.66% compared to a DSSC with a PAniNT counter electrode which exhibits a slightly higher efficiency of 4.13% with a VOC of 775.11 mV, J_SC_ of 9.09 mA cm^−2^, and FF of 58.61% [62]. The improved efficiency, reported when PAniNT is added to the RGOA matrix, is due to the higher catalytic activity of the polymer. Once again, the efficiency of the PANI/RGO composite at an optimum PANI loading (1:1 in this case) (5.47%) almost reaches the PCE reported when the CE was Pt (5.54%) asserting its capability to replace the costly Pt counter electrode in DSSCs (Figure 9).

Inside CPs/graphene or GD composites, the amount of the carbonaceous material within the composite determines the photoelectric performances. Thicker layers provide more catalytic sites and therefore enhance the rate of the reduction processes at the CE. Another parameter important to be followed is the filler factor (FF). When the FF value is higher than the value it has in conventional photovoltaic cells (50%), this parameter describes a successful limitation of the recombination processes at the CP/carbonaceous material interface. The chemical capacitance (Cµ) is used to estimate the electro-catalytic performance of the composite, evaluating its stability and durability. Cµ depends on the active surface area and the pseudo-capacitive charging effect of PEDOT [82,83]. High capacitance value is correlated with lower R_CT_, higher Jsc, and FF, respectively (see Table 3). Related to Cµ is the double layer capacitance (Cdl), used to indicate the catalytic activity and the porosity of the composite structure. In terms of capacitance, the diffusion of I_3_^−^ and I^−^, and the rate of the reduction process, DSSCs with CEs made of composites based on PEDOT: PSS and RGO have very good PCE values, almost reaching the value reported for Pt.

There are only a few occasions when the R_CT_ is not correlated with a high catalytic activity. One of these cases was reported for composites based on PANI and graphene where, although the R_CT_ of PANI/Gr is higher compared to the Pt R_CT_, the PCE values are very close (3.589% for Gr/PANI and 3.976 for Pt) [66]. This could be due to the fact that the Nyquist plot model used for Pt is not appropriate to be used for carbonaceous compounds.

As a general observation based on the analysis of different combinations of graphene and CPs, Chawarambwa, F.L. and co-workers concluded that the addition of a carbonaceous compound reduces the internal resistance of the composite [58]. The reduction process that occurs at CE involve two steps:

I_3_^−^ + 2 e^−^ → 3 I^−^ (1)

3 I_2_ + 2 e^−^ → 2 I_3_^−^ (2)

and the two potential peaks, positive and negative, correspond to the catalytic activity at the CE/I_3_^−^/I^−^ interface and to the activity of the I_2_/I_3_^−^ from the electrolyte/dye interface [84]. With these two parameters, positive and negative potential peaks the list of parameters describing the catalytic performance of CE inside DSSCs is extended.

The list of parameters used to describe the catalytic activity of CE continues with the charge density peaks (Jox and Jred) together with the peak separation (ΔEp) of the reduction and oxidation peak potentials from the redox couple (2I_3_^−^/I^−^). An excellent electro-catalytic behavior is revealed through a high value of current density (Jsc) and low peak separation. According to Dissanayake, M.A.K.L. and co-workers, for the RGO/PANI composite decorated with SnO_2_ nanoparticles a higher value of Jox (1.1 mA/cm^2^) was recorded, compared to RGO (0.53 mA/cm^2^) and RGO/SnO_2_ (0.98 mA/cm^2^ Jox), revealing an improved catalytic activity of the first, followed by a peak separation value for RGO/SnO_2_/PANI close to the one recorded for Pt (0.21 V). A lower ΔEp corresponds to higher electro-catalytic activity in the reduction process of the triiodide ion (I_3_^−^).

There are also inconveniences regarding the quality of composite components. For example, the incomplete reduction of RGO leads to low conductivity involving low charge transfer in the DSSCs [61] and another problem in determining the optimum amount of graphene compound used inside the composite, above the set limit, with the graphene layer overlap causing slow charge transport.

## 4. Synthesis and Vibrational Properties of CPs-CNs Composites as EM for Supercapacitors

### 4.1. Introduction in the Supercapacitors Cells

Recently, numerous studies have been reported on the use of CP-based composite materials, especially PANI and CNs of the type CNTs, graphene, and their derivatives or hybrids, resulted from the combination of the two, in a wide range of applications, in particular, as EMs in DSSCs [16,20,22,24,60,62,68,81] and supercapacitors [85,86,87,88,89,90,91,92,93,94,95,96,97,98,99,100,101]. In the composite configuration, the two components, i.e., CP and the carbon-based material, make their main contribution, the former through chemical stability, mechanical resistance, and redox behavior (PEDOT, PANI) and the latter through conductivity character and a high specific surface area. While in the previous chapter the discussion was mostly focused on DSSCs, this chapter highlights a brief description of the role of PANI/CNs composites as an EM in supercapacitors, where CNs are represented by CNTs and RGO. The description of the two types of electrode composite materials aims to obtain an overview of the aspects that control the synthesis of composites, significantly influencing the electrochemical capacitive performance of the material, the fundamental properties of these EMs being evaluated in terms of specific capacitance and charge/discharge rate.

The explanation for the fact that PANI is the most common CP used in supercapacitor EM lies in its excellent specific capacitance [102], plus the advantages of obtaining relatively simple and economical synthesis by chemical polymerization in an aqueous medium, as well as high stability in air [103]. Its main drawback, correctable by combining with CNs is poor conduction and low stability when used in repeated cycles. The latter is due to the significant changes taking place in the volume of the polymer matrix, being closely related to the processes of doping and de-doping [103]. The combination of PANI and CNs of the type CNTs or graphene or its derivatives is not accidental. Carbon-based materials exhibit very good capacitive behavior (often used as a current collector), high porosity that comes with a large specific surface area, and high chemical and mechanical stability. Thus, by introducing CNs into the polymer matrix, capacitance retention increases during repeated charge/discharge cycles, thus increasing cyclic stability and the charge/ion transfer rate between electrode and electrolyte, as well as the specific capacitance of the device, even at high current densities [104].

The capacitive behavior of the two components, CP and CNs, is different but contributes equally to the value of the final capacitance. PANI and other CPs with redox behavior exhibit pseudo-capacitive or Faradaic behavior where charge storage at the electrode surface occurs through the oxidation–reduction reactions that take place between the EM and the ions in the electrolyte, according to the mechanism reported by Jain D. et al. [86]:

CP → CP_n_^+^ + ne^−^ (p doping); (3)

CP + ne^−^ → CP_n_^−^ (n doping). (4)

In the case of PANI, since it can store charges both in the electric double layer (EDLC) and through the Faradaic charge mechanism, it has been successfully used as an EM for supercapacitors [105]. On the other hand, carbonaceous compounds exhibit both pseudo-capacitive and non-faradaic behavior specific to double-layer capacitors, so called because in non-Faradaic behavior charge storage occurs in the electric double layer at the electrode-electrolyte interface. For storage capacity evaluation, often are recorded (a) cyclic voltammograms at different potential scanning speeds; (b) charge/discharge galvanostatic curves, when a dependence of voltage as a function of time, and (c) for a more detailed study, the Nyquist diagram is made based on electrochemical impedance spectroscopy (EIS) measurements used for the study of charge transfer within the composite, where the main traced parameter is the charge transfer resistance constant which is calculated from the diameter of the semicircle in the Nyquist graph [106]. A low value of charge transfer resistance corresponds to a high value of specific capacitance [107]. Due to the different nature of the two or even three components, in the case of composites, Faradaic and non-Faradaic behaviors occur simultaneously, leading to a greater storage capacity.

For carbon-based compounds, such as graphene, pseudocapacitive behavior is evidenced by a rectangular profile of cyclic voltammograms. This type of behavior is also visible in PANI and other CPs with reversible redox behavior, such as polypyrrole [108,109]. Comparing the cyclic voltammograms profile of the individual components and the composite, we notice a number of differences such as the pseudo-capacitive contribution of PANI [110,111], evidenced by the deviation from the symmetrical triangular shape visible in the case of the composite with CNs. Analysis of charge–discharge galvanostatic (CDG) curves helps to understand the behavior of EM. The profile of the voltage (E)–time (t) curves is triangularly symmetrical in the case of carbon-based materials with ideal capacitive behavior such as CNTs.

The most important parameters of a supercapacitor are the specific energy and power density, which are calculated according to the relationships:

E = 1/2*M* × *Cd* × *V*^2^ and (5)

P = 1/4*M* × (*ESR*) × *V*^2^, (6)

where Cd is the discharge capacitance (F/cm^2^), V is the voltage on the initial range of the discharge curve (excluding IR voltage drop), ESR is the series-equivalent resistance, M is the mass of the EM. ESR includes the interface contact resistance and diffusion resistance of electrolyte ions, as well as particles between electrodes that determine the power density of the supercapacitor [112]. Another indicator of the performance of the EM is the discharge capacitance that is evaluated from the linear portion of the discharge curves, using the relationship:

Cd = *I* × ∆*t* ∆*V*, (7)

where Cd is the discharge capacitance, I is the discharge current, Δt is the time interval over which discharge occurs and ΔV is the voltage variation. The specific capacitance (Cs) is calculated for both the individual EM and the assembly of two electrodes analogous to a supercapacitor and is calculated according to the relationship:

Cs = *I* × ∆*t* ∆*V* × *M* = *Cd*
*M*, for an electrode; (8)

Cs = 2 × *Cd*
*M* for an assembly analogous to a supercapacitor. (9)

Electrochemical impedance measurements evaluate the transport ability of materials. By interpreting the Nyquist graph, important information about the bulk material resistance (Rb) and charge transfer resistance (R_CT_) is obtained. The graph is divided into three zones, the area of low, medium and high frequencies. In the first portion, in the area of low frequencies, the diagram has a step aspect and is an indicator of the capacitive nature of the material [113], while in the middle area, the profile of the graph is usually linear, and at high frequencies it is in the form of a semicircle. In the high-frequency region, the impedance spectrum depends on the load transport process, while in the low-frequency region mass transport processes dominate. For example, in the case of the PANI-Fe_3_O_4_/RGO composite [113], analyzing the spectra through EIS, it is observed that in the case of both RGO/Fe_3_O_4_ and the ternary composite, the charge transfer resistance is low in the high-frequency region and according to the appearance of the graph portion at low frequencies, namely the linear profile, with a slope of about 70°, indicating an ideal capacitive behavior of PANI nanorods [113]. PANI’s nanorod structure has recently been shown to significantly improve the capacitive performance of composites used as EMs in supercapacitors, where the energy storage capacity of a redox material is described by the equation: q t = q electrolyte + q dl + q electrode, where q t is the total charge stored in the electrode, q electrolyte is the charge stored due to the electrolyte, q dl represents the storage capacity of the double layer, and q electrode the charge stored in the active material of the redox electrode [87].

### 4.2. Synthesis and Vibrational Properties of PANI-RGO Composites as Well as Their Performance in Supercapacitors

A very clear application for highlighting the performance of a supercapacitor was made by S. Mondal and his collaborators, who, using the template method, synthesized a ternary composite EM based on PANI and RGO decorated with Fe_3_O_4_ nanoparticles. Mondal S. and colleagues made electrodes from this ternary composite for a supercapacitor that supported the operation of an LED bulb for 30 min [87]. The material synthesized by them had a very good stability, with a capacitance retention of 78% after 5000 cycles. Thus, the transition metal oxide, in the structure of the composite, contributes to increasing its stability and storage capacity. Among the transition metal oxides used in the manufacture of supercapacitors, Fe_3_O_4_ stands out by the large potential window in which it is active, between −1.2 and +0.25 V, benefiting from a theoretical Cs of 2299 F/g plus increased availability, being from natural sources, low cost, and low toxicity, which recommends it for use as an electrode material with pseudo-capacitive properties.

The presence of PANI and RGO in the nanocomposite structure results in the formation of a conductive network due to the π-π conjugation between PANI and RGO-Co_3_S_4_. This phenomenon reduces the electron path length and the diffusion length of the ions in the active material, thus increasing the inner active space, and the possibility of storing more charge weight.

PANI has been used with good results as an EM for supercapacitors, as it can store charges in the EDLC as well as through the Faradaic charge mechanism. In combination with graphene, results on capacitive efficiency of PANI/RGO composite EMs have been reported both in binary combination [88,89,90,91,114] and in ternary composites [87,92,93] and in combination with ionic liquids [94] whose pseudo-capacitive effect is already known [94,115]. Thus, Meriga, V. et al. reported the EM PANI chlorosulfonate/RGO composite with a specific capacitance of 120 F/g in 2015 [91], a value that gradually increased with the addition of transition metal oxide, Fe_3_O_4_, to 283.4 F/g at a current density of 1 A/g [114] and 797.5 F/g at 0.5 A/g with a very good capacitance retention of 92.43% of baseline, after 1000 cyclic voltammograms for the ternary composite with Co_3_S_4_, in which covalent functionalization of RGO-Co_3_S_4_ with PANI takes place [92]. The Cs of the RGO composite also increases when doping RGO with N takes place (282 F/g at 1 A/g) [90], an important aspect in obtaining superior capacitive performance being the porous 3D structure of the synthesized EM, which increases its interaction with the electrolyte facilitating ion transfer at the electrode/electrolyte interface. It has also been shown that the use of 3D structures made of PANI/RGO composite gel leads to greatly improved Cs values, as is the case of the study reported by Wang, Z. et al. [89] on the PANI/RGO composite when a Cs value was recorded of 423 F/g at 0.8 A/g, with a retention of 75% after 1000 cyclic voltammograms. The synthesis morphology of PANI and RGO is also a very important parameter that significantly influences the specific capacitance value of the tested EM; thus, the composite formed by PANI nanowires in combination with 3D type structure of the N-doped RGO leads to a Cs of 385 F/g at 0.5 A/g [116]. Of all of the capacitive values reported, the largest are still maintained on ternary composites with single or double oxides of transition metals, with an additional pseudo-capacitive effect, such as MnO_2_ [117], NiCo_2_O_4_ [93], and others (see Table 4), with a capacitance of 1090.2 F/g at 0.5 A/g and 1235 F/g at 60 A/g, respectively.

In the case of composites based on CPs and CNs, as is the case with PANI/RGO/carbon fibers (CFs), some changes associated with the pseudo-capacitive contribution of PANI show the E-t-profile. Thus, the E-t profile of the composite deposited on CF has two areas: the first area corresponds to a shoulder due to faradaic processes (PANI) and the second area is linear, representing the capacitive process [95].

In the case of the covalent functionalization of RGO with PANI, the reported specific capacitance was 797.5 F/g at 0.5 A/g at a retention of 92.43% after 1000 cycles [92].

A three-dimensional structure of N-doped and PANI-coated RGO has also been reported by Liu, Z. et al. [90] resulting from the in situ chemical polymerization synthesis of PANI in the presence of N-doped RGO using β-MnO_2_ oxidant and polystyrene sacrificial microspheres to create the 3D structure of the RGO network.

Another type of morphology, namely well-separated planar sheets of RGO, without aggregation or association, embedded in the granular matrix of chlorosulfonated PANI, was reported by Meriga, V. and co-workers [91]; this composite material was synthesized by the chemical oxidation polymerization method of ANI in the presence of APS. EMs can also be printed as 3D structures using PANI/GO gel, obtained by the self-assembly method of PANI and GO in a mixture of NMP and water [89], with a capacitance of 423 F/g at 0.8 A/g for the PANI-RGO composite. In order to decrease the toxic compounds used in the reduction process of GO to RGO, Zhao, X. et al. tested tannic acid, a non-toxic compound successfully used for GO to RGO conversion, which influenced the morphology of the obtained composite, namely the PANI microfibrillar network [88]. After 24 h of reaction with tannic acid, the resulting RGO shows a higher specific surface area, a better distribution of PANI fibers on the surface, and a higher specific capacitance compared to the hydrazine-reduced PANI and RGO-based composite. The composite presented above recorded an energy density of 1.68 at 0.5 A/g, and respectively, a power density of 115 KW/kg in the symmetric supercapacitor.

A high capacitance (808 F/g at 53.33 A/g) [98] has been reported for binary PANI/RGO composites, synthesized by two successive processes of self-assembly in a mixture of water and NMP followed by the three-dimensional reduction of the assembly. The highest capacitance value of the PANI-RGO binary composite was reported by Nguyen, Van H. and co-workers (1337 F/g at 15 A/g) [114]. The composite material was prepared using a two-step synthesis method, this being in the form of thin distinct GO sheets with differentiated, wafer-like edges, coated with dendritic PANI nanofibers of 100 nm diameter and micron length. The very low size morphology of PANI is attributed to the chain structure of PANI molecules formed on the surface of RGO sheets [114]. On the other hand, ternary PANI-RGO-oxide composites have been synthesized by a number of methods that significantly influence the morphology of the resulting material. Thus, 3D nano rod-like structures of RGO-doped PANI decorated with Fe_3_O_4_ particles have been obtained by in situ polymerization [87]; 3D structures of the type RGO/PPy/Cu_2_O-Cu (OH)_2_ with very high power densities 8000 KW/kg were obtained by in situ electrochemical polymerization of RGO/PPY on Nickel foam followed by Cu_2_O-Cu (OH)_2_ deposition by chronoamperometry [118]. The granular morphology of PANI-EB in the NiCo_2_O_4_/PANI/rGO composite was reported by Rashti, A. et al., the NiCo_2_O_4_ particles were distributed on the PANI functionalized RGO lattice [93]. The ternary composite was tested in a three-electrode configuration, showing a capacitance value of 1235 F/g at 60 A/g, and in the solid-state asymmetric supercapacitor, the composite acted as cathode and the activated carbon acted as anode, the cell showing a specific capacitance of 262.5 F/g at 1 A/g, with a retention of 78% after 3500 cycles at a working potential of +1.5 V.

Charge storage properties of the composites were tested by GCD. In the case of ternary composite made of RGO, Fe_3_O_4_, and PANI [87] the electrochemical study together with the charge–discharge curves, recorded at 1 A/g, revealed the capacitance behavior of all stages of the composite from binary to ternary, namely for GO, RGO/Fe_3_O_4_ (RGF), and RGO/Fe_3_O_4_/PANI. Therefore, the quasi-triangular shape of the GCD spectrum corresponding to RGO/Fe_3_O_4_ and RGO/Fe_3_O_4_/PANI compared to the ideal triangular GO spectrum shape indicates the presence of two types of capacitances, namely the double-layer capacitance associated with GO and the pseudocapacitance of both PANI and Fe_3_O_4_. The longer charge–discharge time is directly related to an improved charge storage mechanism.

**Figure 10:**
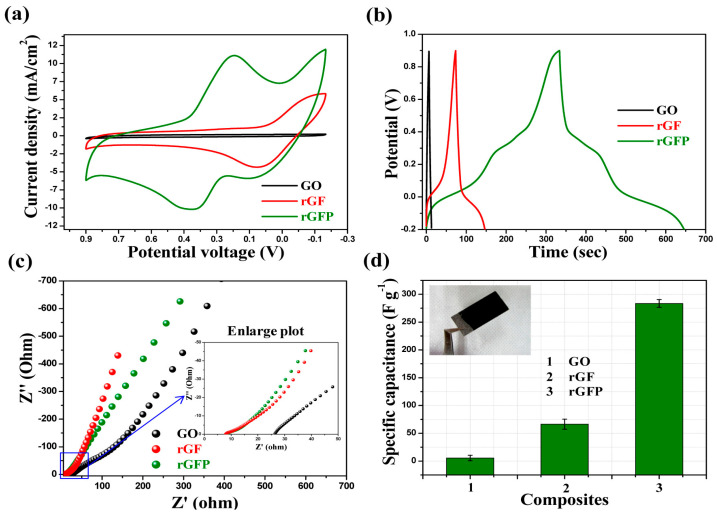
Electrochemical measurement (using the three-electrode system) of synthesized composites: (**a**) CV study (scan rate of 50 mV/s), (**b**) GCD study (at 1 A/g current density), (**c**) EIS study (Nyquist plot, in the 0.1–100,000 Hz range), and (**d**) a bar plot for specific capacitance. All measurements were made in a 0.5 M H_3_PO_4_ aqueous solution [87].

Electrochemical impedance spectroscopic (EIS) studies of GO, rGF, and rGFP composite have been measured in the range 0.1 to 100 kHz. They were performed to evaluate the transport processes within the composites. The impedance spectrum with the two main regions, namely the high-frequency region and low-frequency region is governed by the charge transport process, respectively, by the mass transport process. In the case of RGO/Fe_3_O_4_ and RGO/Fe_3_O_4_/PANI in the higher frequency region (Figure 10c), the semicircle is negligible, revealing the significantly low interfacial charge transfer resistance. In this region, both resistance (8.3 Ω) and charge transfer are low (2.03 Ω).

The slope of the vertical line, at the low-frequency region, is around ~70° indicating the nearly ideal capacitive behavior of the nanorods composite. The specific capacitance (Cs) value for synthesized rGFP ternary composites is high (283.4 F/g) compared to GO (5.5 F/g), respectively, to the binary RGF (66.4 F/g) (Figure 10).

When the EM was represented by RGO-ionic liquid/PANI (RGO-IL/PANI) [94], the reported capacitance was 193 F/g at 1 A/g due to RGO modification with ionic liquid, and a retention capacity of 87% after 2000 cycles at 5 A/g.

Composite materials based on CNTs and CPs, in particular PANI, with applications in the field of supercapacitors have also been reported. These composites generally obtained by oxidative polymerization of ANI in the presence of CNTs [119], respectively, by electrochemical polymerization of ANI on CNTs [120], exhibit three-dimensional structures with superior mechanical and electrical properties, a porous structure, and the ion diffusion property [104] (see Table 5). According to the study conducted by Malik, R. [104], a combination of vertically aligned N-doped CNTs in the form of the CNTs sheets were uniformly coated with a PANI layer, the final material having a core-shell morphology. Thus, the values of Cs reported were 359 F/g and 128 F/g at current densities of 4.95 A/g and 2.42 A/g, respectively, within the solid symmetric supercapacitor with PVA/H_2_SO_4_ type gel electrolyte composite layers between [104]. Vertically aligned N-doped CNTs have three times the specific surface area compared to classical N-doped CNTs and a pore size between 5 and 10 nm which favors PANI deposition and the rapid diffusion of electrolyte ions. According to Haq and co-workers [121], the N-doped centers in CNTs are the ANI polymerization initiation centers. Vertically aligned CNTs were grown on Ni-coated N-doped CNTs sheets by the CVD method from acetylene in an NH_3_ plasma atmosphere [104]. While N-doped CNT substrate sheets (NCNTs) behave as a current collector, vertically aligned CNTs grown on NCNT sheets mediate and accelerate electron transfer from PANI to NCNT layers. The structure retains the core-shell morphology even after 30 cyclic voltammograms and the capacitance retention is 80% after 5000 cyclic voltammograms plus the open porous diode structure which allows ion diffusion while maintaining high ionic conductivity [122,123,124]. Upon covalent interaction between PANI and CNTs, the composite obtained by depositing porous PANI over CNTs with an interconnected pore structure exhibits a Cs of 1266 F/g at 1 A/g, i.e., a retention of 83% after 10,000 cyclic voltammograms [107]. The porous morphology results in more efficient ion transport, faster charge transport due to electron delocalization, and better PANI stability in redox processes. PANI grafting to CNTs leads to the extension of PANI conjugation and thus to increased conductivity up to 3009 S/cm [125]. Charge accumulation and Faradaic redox-reducing properties are improved due to the pore structure and specific surface of the activated MWCNT and PANI-based EM (A-MWCNTs/PANI). Activation of MWCNTs is performed by treatment with HNO_3_ for better dispersion of MWCNTs in the polymer matrix. The Cs value is significantly improved from 42 F/g for A-MWCNTs to 201 F/g after introducing PANI into the EM. This is reflected in the higher current density increase in activated MWCNTs compared to the original ones, the activation positively influencing their porosity. The cyclic voltammograms show an almost rectangular and symmetrical profile, especially at low scan rates, indicating the high capacitive performance of the electrode–electrolyte system.

A frequently encountered problem in the behavior of EMs is maintaining a constant value of Cs at different values of current density. One material that meets this requirement is that reported by Ramana. G.V. and co-workers [119], namely the PANI/CNT composite obtained by oxidative chemical polymerization of ANI, with core-shell morphology and a Cs of 368.4 F/g. According to this study, the functionalization of CNTs prior to composite synthesis allows control of the oxidation state of PANI and can significantly improve the capacitive performance of the material. Another group of researchers, led by Zhou, H. [126], attempted to increase the capacitive performance of a PANI-CNT composite by using electrochemically expanded graphite (ExGP) as a current collector. By reducing the resistance at the EM/current collector interface they demonstrated increased electrochemical capacitance. The EM/current collector couple, mentioned above, has a capacitance of 826.7 F/g, much higher compared to other PANI/CNTs electrodes. The device made from the superposition of two PANI/CNT/ExGP type electrodes with a PVA/H_2_SO_4_ electrolyte layer interleaved is a flexible, solid-state supercapacitor with a power of 7.1 kW/kg at an energy density of 12 Wh/kg with a good cycle stability.

**Figure 11:**
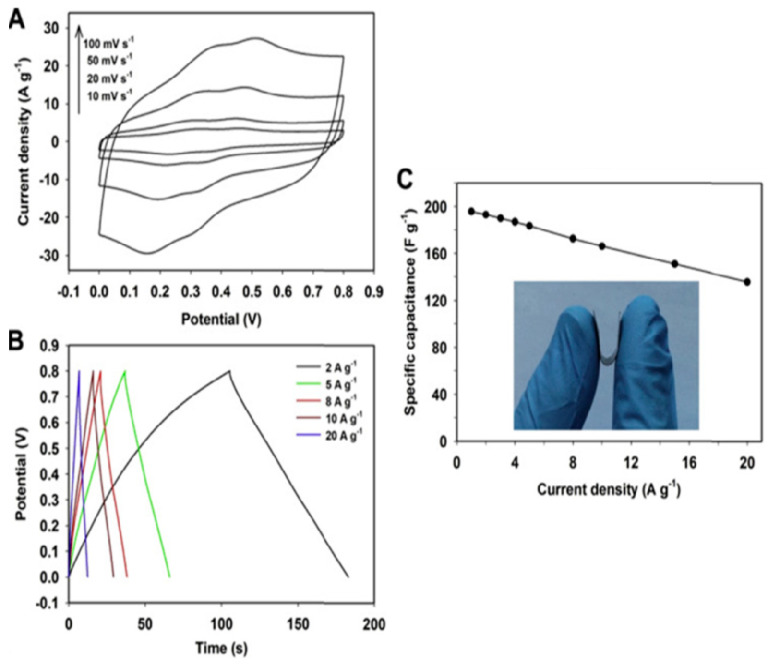
(**A**) Cyclic voltammograms profiles at the scan rates ranging from 10 to 100 mV s^−1^, (**B**) GCD curves at the current densities from 2 to 20 A g^−1^, and (**C**) plot of specific capacitance vs. GCD current density for the symmetric PANI-CNT/ExGP-based supercapacitor. The inset in (**C**) is an image showing the mechanical deformation of the PANI-CNT/ExGP-based supercapacitor [126].

The CNTs used in the composite synthesis were functionalized with -COOH groups by exposure to concentrated acidic medium (H_2_SO_4_:HNO_3_ = 3:1 in volume percent). Due to the expanded graphite substrate, the PANI/CNT morphology is different, thus CNTs are evenly distributed along the PANI fibers, the close contact between the two suggesting the dual role of CNT, as a binding agent and conductive additive, which shortens the distance between the relatively dispersed PANI fibers, facilitating electrical contact between them. The overall appearance is of a core-shell structure, and the length of the CNTs is longer due to the coating with the polymer layer—which increases the specific surface area and thus the number of active centers available in pseudo-capacitive reactions. The formation of the core-shell structure occurs due to the electrostatic interaction between the negatively charged functionalized nanotubes (CNT-COO^−^) and the positive charges along the polymer chain of PANI. The higher specific capacitance of PANI/CNTs/ExGPs compared to PANI/ExGPs is evidenced by a larger cyclic voltammograms area and more intense redox maxima, respectively (Figure 11).

**Figure 12:**
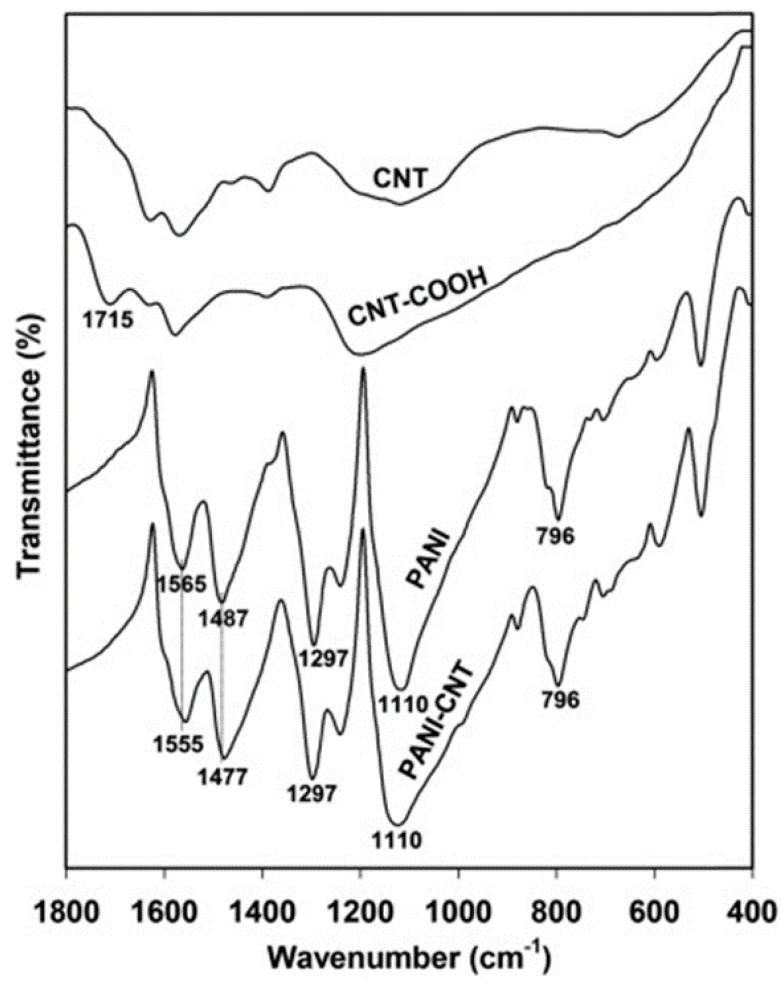
FT-IR spectra of CNT, CNT-COOH, PANI, and PANI-CNT.

Figure 12 shows the FTIR spectra of CNT, carboxylated CNT, PANI, and PANI-CNT. The FTIR spectrum of CNTs-COOH compared to CNT exhibits an additional peak (1715 cm^−1^), due to C=O stretching vibration in carboxyl groups [127], which is a proof that carboxyl groups were introduced into CNT during the functionalization process. The -COO^−^ groups on the surface of CNTs neutralize the positive charges in the oxidized PANI chain, during electrochemically oxidative polymerization, thereby forming the CNT@PANI core-shell structure mentioned above. The PANI-CNT spectrum is almost similar with PANI spectrum the only difference being that the typical C=C bonds in PANI located at 1565 and 1487 cm^−1^ are red-shifted to 1555 and 1477 cm^−1^ in the FTIR spectrum of the composite, due to the interaction between CNT and p-electrons of PANI chains [128].

CNTs could be prepared before interaction with PANI by the ball-milling process and subsequent exposure to acid solution. The resultant composite, MWCNT-PANI, exhibited a capacitance of 837.6 F/g at 1 mV/s [129]. The increase in anodic and cathodic current densities with increasing scan rate indicates a good material operating rate, which is accompanied by the large cyclic voltammograms area relative to that of the CNT indicating a large pseudo-capacitance of the electrode. Due to the electrostatic interactions of the positive charges in the PANI chain and the anions in the solution, floccules are formed that allow the ions to access their surface, reducing the distance between them and the PANI, favoring the charge/discharge processes. A special type of PANI/CNT composites are those in which the CNTs are open at one end (so-called partially unzipped CNTs), and play a role in favoring the access of ions from the electrolyte to the EM during charge–discharge processes, contributing decisively to increasing the mechanical strength of the EM. CNTs can be opened by several methods, such as chemical interaction with acids [130], intercalation and exfoliation of lithium ions [131], catalytic processes [132], electrical [133], and physical–chemical methods [134]. The CNT unbonding process consists of the longitudinal splitting of the nanotube, a procedure that results in one or more layers of graphene or a combination of inner tube and graphene nanosheets, depending on the number of CNT walls. While the first mentioned methods used to unzip the CNTs involve the use of chemical compounds, the last two are cleaner methods to shape the final product, namely graphene nanoribbons (GNR). While the electric method as it says uses the electrical current to tailor a graphene sheet in high vacuum conditions, the physical–chemical method assumes the coverage of CNTs casted on substrate (Silicon wafer) with a thick film of poly methyl methacrylate (PMMA), followed by heat treatment to strengthen the CNTs-PMMA structure. After that, it follows the exfoliation of the resulted CNTs-PMMA film from the substrate using the KOH solution. The final step represents the etching process which occurs by exposing the sample to 10 W Argon plasma, for various periods of time, resulting in one or multi-layered GNRs or a combination between GNR and CNTs depending of the CNTs number of walls.

**Table 5:** PANI-CNT composites, synthesis methods, morphology, and capacitive performance.

Composite	Morphology	Synthesis Method	Cs (F/g)	Ref
PANI/CNTs	Homogeneously co-dispersed open tubes together with graphene nanostructures resulting from the process of opening tubes coated with a uniform polymer layer *	In situ oxidative polymerization	762 with 81% retention after 1000 cyclic voltammograms	[85]
A-MWCNTs/PANI	Thick bundles of CNTs	In situ polymerization	248 at 0.25 A/g, and 99.2 F/g at 5 A/g	[103]
PANI/NCNT (CNT dopat cu N)	Vertically aligned nanotubes grown perpendicular to horizontally aligned CNTs coated with a uniform layer of polymer, observed by increasing the diameter of the CNTs	Electrochemical deposition	359 at 4.95 A/g with 82% retention at 46.87 mA/cm^2^	[104]
Porous PANI/CNTs	Compact morphology	Chemical grafting by Ani interaction with -NH_2_-functionalized CNTs	1266 F/g at 1 A/g, 83% after 10000 cyclic voltammograms	[107]
CNT-PANI	Shell-core structure	Oxidative chemical polymerization	368.4 F/g at various current densities	[119]
Composite expanded graphite (ExGP)/PANI-CNT	Interconnected fiber microstructures interleaved with CNTs	Electrochemical co-deposition	826.7 F/g	[127]
PANI/CNT	Polymer deposition on the surface of CNTs	In situ chemical polymerization	837.6 F/g at 1 mV/s, 68% after 3000 cyclic voltammograms	[129]
PANI/MWCNTs	Nanofibrous structure	In situ chemical polymerization	554 F/g at 1 A/g	[135]

* Outer tube of partially destroyed MWCNTs transformed into graphene nano-ribbons and core-like inner tube in the PANI composite; a decreased diameter of PANI fibers was observed suggesting the coating of hybrid MWCNTs with a uniform polymer layer.

## 5. Conclusions

Composite materials used as CEs after being deposited on FTO substrate present different features depending on the CPs type, the carbon nanostructures inside the composite, the interaction between both components which is directly related to the type of synthesis, and so on. Composites based on CNTs and CPs were prepared through different methods, starting with CNTs deposited on FTO or ITO substrates, followed by polymerization of monomers, precipitation of the polymer on top of CNTs, or by one-pot electrochemical synthesis from a CNTs dispersion mixed with a monomer solution. The only one which provides good control of the thickness and uniformity of the grown layer is the electrochemical method. Excellent results were also reported when the CVD technique was used to synthesize polymers on top of CNTs. Through CPs-CNTs composites, a special morphology is the honeycomb-like structure obtained on the CNTs substrate using a PMMA template (in the case of PPy and PEDOT composites with MWCNTs), when the CPs is electropolymerized on top.

Inside CPs/CNTs composites, CNTs play the role of template leading to porous nanostructured wires but the CNTs were wrapped in CPs film through π-π coupling (as in the case of CNTs-PEDOT composite). The use of aligned structures or well-ordered CNTs have been reported to enhance the PCE value of DSSC by decreasing the resistance and increasing the conductivity.

An innovation in the CE materials field was also the incorporation of CNTs inside a gel electrolyte, to improve the DSSC PCE performance. The purpose was achieved through the significant contribution of CNTs to enlarge the SA of the composite, increasing the conductivity and the Jsc and thus providing a high catalytic activity. Depending on the synthesis method used for their preparation, CNTs were reported as covalently (PPy-CNTs) and non-covalently functionalized with CPs. The first were obtained through the reflux technique followed by in situ polymerization and the second through all the other methods mentioned above.

Non-covalent functionalization of CNTs with CPs was revealed in the changes observed in the IR and Raman spectra of the individual component and on the composite. Due to the interaction between π-π bonds from the polymer structure and MWCNTs, the bands assigned to the C=C/C-C stretching vibration shift (case of PPy chains) to lower frequencies. The shift to lower frequencies is due to short polymer chains formation and could be accompanied by a decrease in the IR main band intensity (bands that could be associated to C-H, C-C, and N-H or C-N, depending on the CPs backbone structure).

Part from the interactions specific for non-covalent functionalization, namely the electrostatic interaction, which takes place between the donor (usually aromatic or quinoid ring from the CPs) and the acceptor (usually the CNTs), determines a fast movement of charges inside the composite enhancing its conductivity. CNTs wrapped within the CPs could create a transport network for electrons improving the cathodic reaction of the redox couple I_3_^−^/I^−^ (in the case of FMWCNTs-PPy composite).

When CNTs are covalently functionalized with CPs, new bonds are formed, namely covalent bonds, between CPs and CNTs, which are formed between –NH- from CPs and –C= from SWCNTs (inside PANI-SWCNTs). Through these covalent bonds, the charge transfer between the components of the composite is accelerated.

In the case of CPs/graphene or GD composites, all of the synthesis methods, namely mechanically mixing graphene and CP, the deposition of a polymeric film on top of graphene through in situ chemical polymerization, and the organic sol-gel route, have in common the individual deposition of the polymer and the graphene/GD layer. This is the reason why the morphology of the resulted composites is close to a sandwich structure. Regarding CPs/GD composites, GD (for example GO) provides appropriate support for the nucleation and polymerization of the monomers, precursors of CPs; meanwhile, the CPs ensures the homogenous dispersion of GD. Therefore, the combination of GD with CPs lead to the formation of composite materials as a promising candidate for a CE in the DSSC device. Special structures of CPs/GD composites play an important role as CEs in DSSC. The aerogel structure obtained by the organic sol-gel route, followed by freeze-drying, presents a high surface area and excellent catalytic activity towards the reduction of I^−^/I_3_^−^ in the electrolyte. The three-dimensional structure of graphene aerogels provides special properties such as a high electric conductivity and large volume of pores, facilitating the charge transport and the mass transfer of redox species.

At the interface of both CPs and GD the non-covalent functionalization is revealed through interactions such as π-stacking, hydrogen bonding, electrostatic, and donor–acceptor take place. The exchange of charge carriers between CPs and GD contributes actively to the electrolyte regeneration process providing an appropriate number of electrons. The weak bounded electrons which travel from CPs to GD (RGO) also represent a significant source of electrons for regenerating the electrolyte redox couple I^−^/I_3_^−^. Regarding the ternary composites, namely metallic NPs/CPs/CNT or GD, the presence of metallic catalytic nanoparticles on the composite surface has been proved to contribute significantly to an improved PCE (Cu/PPy/MWCNTs, RGO/SnO_2_/PANI). Not only at the surface but also inside the GD, oxide metallic NPs lead to increased porosity by creating interconnections and pathways between the components of the composite. This fact leads to enhancement in the reduction rate of I_3_^−^ at the CE (redox process) and therefore efficiency improves considerably.

The best performance was reported for CPs/GD composites obtained through a mechanical mixture of graphene and PEDOT: PSS with a reported PCE value of 9.57%, followed closely by a modified GD with SnO_2_-PANI composite with 8.68% PCE. In the case of CPs/CNTs composites, the best results were reported on PEDOT-MWCNTs, 9.07% followed by Cu-PPy-CNT, 7.1% PCE.

Composites based on PANI and CNs shaped as CNTs and RGO can also be used as EM in supercapacitor devices due to the combination of the superior mechanical and electrical properties of CNTs and RGO, ion diffusion properties, as well as both the conductive and pseudo-capacitor character of CP. Capacitive performance requires that the EM has a high capacitance value and a cyclic retention as close as possible to the initial capacitance value. Within the composite, both components contribute to the increase in Cs, on the one hand the CNs acts as a current collector, with a high specific surface area and superior mechanical strength that determines the cyclic stability of the EM into whose structure it enters, and on the other hand the CP is the active component, responsible for ion diffusion and electron transfer due to the extensive delocalization that takes place between the CP and the CNs, either RGO or CNTs. From the series of the CPs/RGO composites, the best capacitive performance has been reported for ternary composites with oxides of transition metals, which exhibit an additional pseudo-capacitive effect, e.g., MnO_2_, NiCo_2_O_4_, Co_3_S_4_. As important as a high Cs value is, the cyclic retention should be as close as possible to the initial value after exposing the EM to several charge/discharge cycles. The different composite morphologies obtained from combinations of CNs, respectively, e.g., RGO with CP, are also an important indicator in predicting capacitive performance.

In the case of the PANI-CNTs composites, the morphologies range from compact to porous structures, from core-shell structures to nanofibers, with a capacitance value of 1266 F/g at a current density of 1 A/g and a retention of 83% after 10,000 cycles reported for the composite with CNTs covalently functionalized with PANI, demonstrating a higher cyclic stability in the case of CNTs composites as opposed to those with RGO but also a significant contribution of the type of functionalization within the composite. Very good results were also obtained by reducing the resistance at the electrode–electrolyte interface by introducing an exfoliated graphite substrate acting as a current collector.

## 6. Outlook

In terms of conversion efficiency, many leaps have been made, starting from around 4.04% PCE [136] to 9.07% reported in the case of composites based on CPs and CNTs [54].

The biggest issue remains to limit the recombination processes from the TiO_2_/electrolyte interface to obtain a more efficient conversion. Energy is lost when the photons, with higher energy than the threshold value, dissipate the excess of energy releasing heat instead of generating more electrons of low energy, the so-called thermal/thermalized electrons originating from the energy of a single absorbed photon.

To limit the recombination, many ways could be tried, such as, for example, to recover the lost energy, which remains unabsorbed and unconverted, to capture and reabsorb it through the same system, or use an additional system for a more efficient conversion. This could be achieved further by using radiation-absorbent materials. These kind of materials have a band structure similar to quantum dots (for example, PbSe, known to produce, at high yield, multiple excitons from a single absorbed photon) or quantum wells and to find a way to extract and transfer photogenerated carriers from the quantum dot structure to produce electricity in the external circuit.

Another issue regards the distance between the photoanode and the electrode. Inside DSSCs, light conversion is assured by the TiO_2_ particles network which collects the charge carriers (e^−^). The absorption of light is performed by the molecules of dye deposited on the nano-porous surface of TiO_2_ and by the interfacial contact distance, between the photoanode and the CE, kept low by the help of the electrolyte, sandwiched between them.

Eligible materials that could play the role of the electrolyte could be materials with mini-bands or intermediate bands such as the organic CPs or composites. Inside these kinds of materials, different energies of incident photons could promote the absorption at different isolated energetic levels, providing different voltages. A liquid CP should penetrate the porous structure of the solid photoanode and collect other types of carriers to complete the circuit from inside the cell.

With regard to supercapacitors, the main problem which is now in progress is the stability during cyclic exposure which could be diminished by using current collectors, not necessarily metallic foil but compounds with fibrous morphology eventually covered with thin metallic film. Another distinct drawback addresses the leaching of the electrolyte from the devices, a drawback which could be managed by encapsulation of the entire storage device in order to improve the Cs and to avoid the degradation of the EM through delamination. Apart from nano-carbonic materials, which represent the best candidate for EM in supercapacitors due to their high compatibility, cyclic retention, and resistance to corrosion, nano-cellulose may be a future good option due to its property of uniformity when shaped in thin films together with mechanical resistance.

There are also other types of polymers involved in energy storage as a part of the supercapacitor devices, namely the biopolymers such as alginate or chitosan which in combination with inorganic materials, such as molybdenum acid salts or manganese dioxide (electrolytically synthesized) lead to excellent charge storage properties [137,138]. The hybrid materials based on molybdenum acid salts have the general formula MMoO_4_ (where M is a divalent cationic metal such as, for example, Co, Mn, Ni, Ca, Fe, Cu, or Pb). They have the advantage of easy synthesis, excellent efficiency, cycling stability, and they are environmentally friendly. For instance, cobalt molybdate exhibits a Cs of 1558 F/g together with an excellent cyclic retention, while chitosan-modified CoMoO_4_ exhibits a capacitance of 135 F/g at the current density of 0.6 A/g and an energy density of 31 W h/kg, with a cyclic retention of 60% over 2000 cycles [139,140]. There are also other combinations of molybdenum salts within composites used as supercapacitors materials, such as NiMoO_4_ and NiCo_2_O_4_ with a Cs equal to 2474 F/g [141], while the MnMoO_4_/graphene hybrid composite shows a Cs of 364 F/g in the three-electrodes configuration [142]. All of these materials are the future trend in charge storage devices. The combination between biopolymers [137,138] and molybdenum salt hybrids is a perfect match due to the strong adhesion of the molybdate to the polymer network [143]. Similar to chitosan, another widely used biopolymer is alginate [137]. In combination with electrolytic manganese dioxide (namely EMD), this delivered five-times-higher capacitance than pristine EMD (487 vs. 94 F/g) at 1 mA/cm^−2^ in highly alkaline aqueous electrolyte. Coupled with activated carbon, the EMD composite exhibited a capacitance of 52 F/g and a cyclic retention of 94% over 5000 cycles [137]. Therefore, considering the trend towards more natural materials as EMs in supercapacitors, the composites based on biopolymers and hybrid materials based on molybdenum acid salts represent a future alternative to be exploited in this field due to their high capacitance and long cyclic stability.

## Figures and Tables

**Table 2:** Synthesis and CE performance parameters (CE, FF, Jsc, and R_CT_) of the CNTs/CPs composites.

Composite CPs/CNTs	Synthesis	PCE (%)	FF	J_sc_ (mA cm^−2^)	Rct (Ω × cm^2^)	Ref.
(a) CNTs/PPy (b) CNTs/PANI (c) CNTs/PEDOT	Electrochemical synthesis	6.82; 7.01; 7.2	0.69	13.73; 13.92; 14.11	1; 7.43; 7.5; 7.51	[16]
MWCNT-PEDOT: PSS	Physical mixing	6.1%	59.8	12.9	-	[18]
* h-PEDOT/MWCNTs	Electropolymerization	9.07	0.67	17.09	0.19	[53]
PPy/SDS/CNTs	Electrochemical polymerization	PPy-SDS-CNT 6.15	PPy-SDS-CNT:58.69	15.47	0.19	[19]
PPy/MWCNT/FTO	Electrochemical polymerization	1.67%	0.53	5.44	-	[17]
(A) Cu-PPy-CNT (B) PPy-CNT	Electrodeposition method	(a) 7.1% (b) 5.49	(a) 0.696 (b) 0.682	(a) 2.35 mA/cm^2^ (b) 10.27	(a) 4.31 Ω × cm^2^ (b) 5.29	[21]
PPy-SWCNTs	Chemical polymerization	8.3%	0.71	15.68	8.15	[36]
PANI-SWCNTs	Electropolymerization	front ill **: 7.07%	0.53	17.5	0.18	[24]
PANI/SWCNT/ZnO nanorods	Polymer precipitation top of MWCNTs	-	-	-	-	[22]
PANI/SWCNT/ZnO	One-pot electrochemical synthesis	PS: 3.16 and PSZ: 3.81	PSZ (PANI-SWCNT-ZnO): 56	PSZ: 9.59	PSZ: 10.10	[20]

* honeycomb-like structure. ** illumination.

**Table 4:** PANI-RGO composites, synthesis methods, morphology, and capacitive performance.

Composite	Morphology	Synthesis Method	C_s_ (F/g)	E (Wh/kg)	P (KW/kg)	Ref.
**Ternary composite rGO/Fe_3_O_4_/PANI**	3D Nanorods of PANI doped with RGO decorated with Fe_3_O_4_	Template method	283.4	47.7	550	[87]
**RGO/PPy/Cu_2_O-Cu (OH)_2_**		Electrochemical polymerization	997 la 10 A/g,	20	8000 19,998.5	[118]
**PANI-RGO**	Globular or nano rods PANI on the surface of the RGO	In situ oxidative polymerization Pe RGO	797.5 F/g la 0.5 A/g, 92.43% after 1000 cycles			[92]
**PANI-RGO**	3D Porous composite PANI/RGO, with a specific surface of 228 m^2^/g	Oxidative polymerization	420 F/g la 0.2 A/g, 80% after 6000 cycles at 2 A/g	9.3 for symmetric supercapacitor	0.1	[96]
**PANI-RGO**	RGO sheets randomly aggregated and closely linked together, uniformly coated by PANI nanofibers	Polymerization method surfactant-assisted	444 F/G la 0.6 A/g	13.36 W × h/kg	1.03 kW/kg	[86]
**3D composite of the type RGO doped with N-PANI**	PANI nanowires	In situ chemical polymerization	282 F/g la 1 A/g, 64.5% after 1000 cycles	-	-	[90]
**PANI-RGO**	Planar sheets of RGO, granular matrix of chlorosulfonated PANI	Chemical oxidation	120 F/g for PANI-RGO, 94% RGO	-	-	[91]
**PANI/RGO**		3D structure printing	1329 mF/cm^2^ 423 F/g at 0.8 A/g	-	-	[89]
PANI- tannic acid -RGO	Micro-fibrillary network of PANI	In situ oxidative chemical polymerization	268.5 F/g t 10 mV/s	1.68 la 0.5 A/g in symmetric supercapacitors	115	[88]
PANI-RGO- carbon fiber, ternary composite	Aggregate sheets with fine layers of PANI	Electrochemical method	430 F/g at 10^−3^ Hz	-	-	[95]
RGO/CNT-PANI	Fiber-shaped electrodes, skeleton/skin structure	GO reduction, PANI electrodeposition	193.1 F/cm^3^ at 1 A/cm^3^, 80.6% after 2000 cycles	0.98 (mW × H/cm^3^)	16.25 (mW/cm^3^)	[97]
NiCo_2_O_4_/PANI/rGO	Granular shape of PANI emeraldine base	Chemical polymerization	1235 F/g t 60 A/g, 78% after 3500 cycles	45.6 W × h/kg	610.1 kW/kg	[93]
PANI-RGO	Composite gel with 3D structure, porous	Self-assembly followed by a reduction process	808 F/g at 53.33 A/g	-	-	[98]
RGO-PANI	Nano-rods	Chemical polymerization	524.4 F/g at 0.5 A/g, 81.1% after 2000 cycles at 100 mV/s	-	-	[99]
PANI-RGO	Fibrillary morphology	In situ chemical polymerization	250 F/g	-	-	[100]
RGO-ion liquid/PANI (RGO-IL/PANI)	Excellent flexibility	In situ chemical polymerization	RGO-IL: 193 F/g at 1 A/g with 87% after 2000 cycles at 5 A/g	24.1	501	[94]
RGO-PANI	Dendritic nanofibers of PANI	Chemical polymerization	1337 F/g at 15 A/g, 81.25% after 5000 cycles	-	-	[114]
PANI-RGO- nanocellulose	Fibers	Chemical synthesis	79.71 F/g	Power density from 110.45 to 50.65 W/kg	-	[101]

## Data Availability

Not applicable.
